# The Place of RNA in the Origin and Early Evolution of the Genetic Machinery

**DOI:** 10.3390/life4041050

**Published:** 2014-12-19

**Authors:** Günter Wächtershäuser

**Affiliations:** 209 Mill Race Drive, Chapel Hill, NC 27514, USA; E-Mail: gwmunich@bellsouth.net; Tel.: +1-919-942-5943; Fax: +1-919-929-9383

**Keywords:** last universal common ancestor (LUCA), thermal evolution, ligand-accelerated catalysis, peptide cycle, pre-ribosome, Wong theory, urzymes, all-purine RNA, trionucleic acid, flow setting

## Abstract

The extant genetic machinery revolves around three interrelated polymers: RNA, DNA and proteins. Two evolutionary views approach this vital connection from opposite perspectives. The RNA World theory posits that life began in a cold prebiotic broth of monomers with the *de novo* emergence of replicating RNA as functionally self-contained polymer and that subsequent evolution is characterized by RNA → DNA memory takeover and ribozyme → enzyme catalyst takeover. The FeS World theory posits that life began as an autotrophic metabolism in hot volcanic-hydrothermal fluids and evolved with organic products turning into ligands for transition metal catalysts thereby eliciting feedback and feed-forward effects. In this latter context it is posited that the three polymers of the genetic machinery essentially coevolved from monomers through oligomers to polymers, operating functionally first as ligands for ligand-accelerated transition metal catalysis with later addition of base stacking and base pairing, whereby the functional dichotomy between hereditary DNA with stability on geologic time scales and transient, catalytic RNA with stability on metabolic time scales existed since the dawn of the genetic machinery. Both approaches are assessed comparatively for chemical soundness.

## 1. Introduction

Metabolism, genetics and cellularity of life are closely interrelated. Genetics imposes catalytic control on the metabolism, yet its components derive from this very metabolism. The cell structure derives from the genetically controlled metabolism, yet it is required to hold genetics and metabolism together. How did this interdependence come about? Did a primordial metabolism invent its own genetic control or did a primordial genetic system conquer its metabolic resources? How did cells enter the picture? And what is the place of RNA and peptides/proteins in all that? We use a stepwise approach. First we reconstruct in the backward direction (retrodict) the characteristics of the last universal common ancestor (LUCA) beginning with extant genomics. Thereafter we interpolate between LUCA on the one side and competing theories of the origin of life on the other side. This evolutionary interpolation provides us with competing accounts of early evolution. We evaluate these accounts along Popperian lines for their relative explanatory power [1], their compatibility with the logic of the chemical situation, and their aptitude to grow in comprehensiveness and coherence by the integration of independently developed evolutionary theorems based on extant biochemistry and genetics. Given the long time frame of evolution of >4 billion years and the complexity of the extant biosphere as *explanandum*, a comprehensive, coherent account will require more or less risky speculations, broad in scope and incomplete in detail, that are ahead of their experimental time. When we can demonstrate an orderly course of progress, each advance building on previous advances and giving rise to further advances, then we shall know that we are on the road to a mature science.

## 2. The Place of RNA in LUCA

The method of characterizing LUCA by homology of individual genes between the domains Bacteria and Archaea (disregarding chimaeric Eukarya) yields typically ambiguous results due to gene losses and gene transfers and even more intensely so the deeper we go in the tree of life [2] (Woese theorem). We therefore have to resort to comparing larger integrated contexts with multifunctional ramifications for whole organisms. One of these contexts is genome organization.

**LUCA Consensus Gene Cluster.** Comparison of gene order in bacterial and archaeal genomes reveals the existence of gene clusters (mainly for transcription/translation) of conserved gene order but differing cluster lengths, whereby variations of gene order and cluster length correlate well with accepted taxonomic groupings. These gene clusters, scattered throughout very large genomes, are unlikely to be accidental or the result of convergence. Lateral gene cluster transfer can also be excluded. It would cause an overwhelming proportion of hybrid machineries of transcription and translation with multiple impairments and the combined effects of their functionally impaired protein products would lead to dysfunctional cells. Therefore, gene order conservation is the only remaining explanation. Alignment of these gene clusters (using 16S rRNA phylogeny as *prima facie* guide) produces alignment packages that fit together with overlapping fringes like pieces of a puzzle. Using gene order conservation for polarization this permits the construction of extended ancestral consensus gene clusters that increase in length with increasing depth of the corresponding nodes in the phylogenetic tree. In this fashion we arrive at hypothetical consensus gene clusters for the last common ancestors of all Bacteria (LBCA), all Archaea (LACA), and LUCA (Table 1) [3]. The original publication [3] was based on 19 microbial genomes and therefore has a sampling bias. Now, however, it has support by a great number of genomes of the domains Bacteria and Archaea that have become available later. Importantly, it is also in agreement with later independent analyses [4,5,6]. The gene cluster of LUCA may have actually included further genes that were later lost by reductive evolution, a phenomenon that is evidenced by the Euryarchaeota [7]. Moreover, gene sequence homology analysis will fail to recognize certain segments of LUCA gene order due to frequent mutational saturation. This renders the fact that the gene homologies of Table 1 since the time of LUCA are still recognizable all the more astounding.

**Table 1:** Updated consensus gene clusters (designated as gene products) of the last common ancestors of Bacteria (LBCA), Archaea (LACA) and last universal common ancestor (LUCA) (double line = cluster break).

LBCA	SecE	NusG	L11p	L1p	L10p	L12p	—	Rpoβ	Rpoβ’	L30e	—	S12p	S7p	EF-G
LACA	SecE	NusG	L11p	L1p	L10p	L12p	RpoH	RpoB	RpoA	L30e	NusA	S12p	S7p	EF-2
LUCA	SecE	NusG	L11p	L1p	L10p	L12p	—	Rpoβ	Rpoβ’	L30e	—	S12p	S7p	EF-G
LBCA	EF-Tu	S10p	L3p	L4p	L23p	L2p	S19p	L22p	S3p	L16	L29p	—	S17p	L14p
LACA	EF-1α	S10p	L3p	L4p	L23p	L2p	S19p	L22p	S3p	—	L29p	Rpp29	S17p	L14p
LUCA	EF-Tu	S10p	L3p	L4p	L23p	L2p	S19p	L22p	S3p	—	L29p	—	S17p	L14p
LBCA	L24p	—	L5p	S14p	S8p	L6p	—	—	L18p	S5p	L30p	L15p	SecY	adk
LACA	L24p	S4E	L5p	S14p	S8p	L6p	L32e	L19e	L18p	S5p	L30p	L15p	SecY	adk
LUCA	L24p	—	L5p	S14p	S8p	L6p	—	—	L18p	S5p	L30p	L15p	SecY	adk
LBCA	map	IF-1	L36	S13p	—	S11p	S4p	Rpoα	L17	—	L13p	S9p	—	S2p
LACA	7 genes		—	S13p	S4p	S11p	—	RpoD	—	L18e	L13p	S9p	RpoN	S2p
LUCA	—	—	—	S13p	—	S11p	—	Rpoα	—	—	L13p	S9p	—	S2p

**LUCA Pre-Cell Organization.** Based on the absence of cell wall commonalities between the Bacteria and Archaea it has been proposed that LUCA existed as wall-less precells [8,9,10] (Kandler theorem I). The LUCA precells must have been bounded by lipid membranes as evidenced by the fact that the consensus gene cluster comprises genes for the main subunits (SecE, SecY) of protein translocase, which is lipid membrane-embedded [3]. It has been proposed that the domains Bacteria and Archaea owe their segregated existence to the opposite chirality of their lipids [11,12] (Koga theorem). The wall-less LUCA precells must have undergone frequent fusions [3], as evidenced by *Thermococcus coalescens* [13], and they may have divided by budding, like L-form bacteria or mycoplasms [14,15] (Kandler-Errington theorem). Lateral gene transfers are a consequence of these cellular mechanisms and caused global uniformity of life as represented by the common stem of the universal tree of life. The close structural kinship of bacterial and archaeal core lipids suggests that LUCA had a membrane of chiral phosphatidyl lipids [11,12]. By further considering that many LUCA enzymes had no enantioselectivity we conclude that LUCA phosphatidyl lipids were synthesized as racemate [16]. The resulting heterochiral membranes must have had a sufficient thermodynamic stability to support a long evolution, during which the precells acquired the multi-component genetic machinery [16,17].

A separate hypothesis posits that the LUCA membrane underwent thermodynamically favored intracellular segregation into a patchwork of homochiral membrane domains of the overall racemic membrane. Moreover, it is proposed that the frequent fusions and fissions of the precells caused intercellular segregation into two enantiomerically distinct subpopulations of precells with a predominance of one lipid enantiomer over the other, *i.e.*, each with an enantiomeric excess. This intercellular segregation is thermodynamically driven by decrease of line tension between the membrane domains. While the lipids are continuously synthesized as racemate they are also continuously segregated with the result of a steady state enantiomeric excess. In agreement with the Koga theorem, it has been proposed that these subpopulations served as placeholders for the later emergence of the domains Bacteria and Archaea [16]. This hypothesis is testable by comparing a membrane of a lipid racemate with homochiral membranes of the two enantiomers that are comprised by the same racemate, and it has indeed been confirmed for vesicles of 2-methyldodecanoic acid [18]. Other experimental results [17] are unfortunately inconclusive, because highly derived bipolar lipids of hyperthermophilic Archaea were combined with monopolar lipids of mesophilic Bacteria or Eukarya in terms of the ability of the resulting mixed membranes to operate kinetically as leakage barrier.

**LUCA Genome Organization.** The rigidly conserved gene order of LUCA implies high genome stability that can only be afforded by covalently circularized chromosomes of double-stranded DNA. A LUCA RNA genome would be instable due to the 2’-OH groups, which lead to intramolecular self-destruction, notably at elevated temperature and pH. Thermally stable RNA is restricted to a narrow sequence space [19] (Maurel theorem) that is incompatible with the freedom of sequence information required for an RNA genome. Therefore, LUCA must have exhibited the extant DNA/RNA dichotomy with thermo-stable DNA in charge of heredity and thermo-instable, self-destructing RNA in charge transient catalysis. DNA has a half-life on geologic time scales, while catalytic mRNA has a half-life on metabolic time scales.

The absence of rRNA genes from the LUCA consensus gene cluster suggests a chromosomally separate rRNA operon. Metabolic genes are also absent and would have also been encoded on separate chromosomes. They would have sorted out differentially in different chemical habitats, thus rendering the overall LUCA precell population metabolically multi-phenotypical [8,9,10] (Kandler theorem II). The extremely conserved order of genes for the gene expression machinery is incompatible with the order-out-of-chaos proposal that the genomes of the domains Bacteria and Archaea “crystallized” from a set of individual genes [2]. Rather, it supports the notion of an order-out-of-order evolution from a rigid primordial state towards ever-greater looseness.

**Figure 1:**
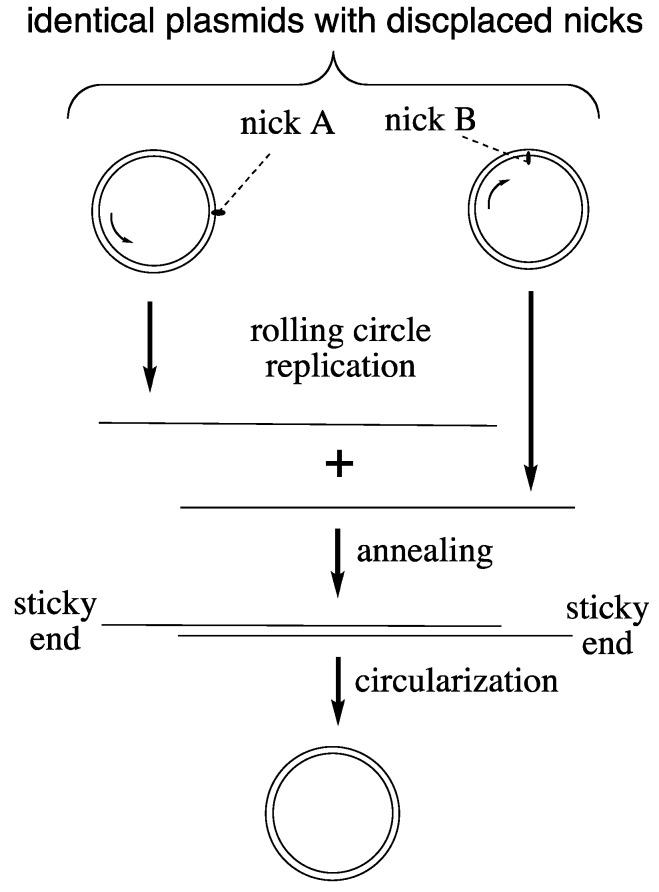
Hypothetical rolling circle replication of LUCA plasmids.

**LUCA Genome Replication.** DNA polymerases of Bacteria and Archaea lack similarity [20] from which it has been concluded that modern DNA replication emerged twice independently (Koonin theorem). Moreover, DNA replication genes are absent from the LUCA consensus gene cluster. Yet there is stunning similarity of the overall replication process across all three domains, and genes for several ancillary replication factors show inter-domain homology [20]. For resolving this inconsistency we broaden our view to include the replication of small, circular, double-stranded DNA plasmids. In the domains Bacteria and Archaea they occur in large copy numbers, practicing a replication mechanism of their own: rolling circle replication [21]. A primitive version of this mechanism (Figure 1) may have operated in LUCA on plasmid-sized circular chromosomes [16,22,23]. We simply assume that nicks occurred in both strands in displaced positions for starting bidirectional replication. The nicks relax the chromosome, which obviates the need for a helicase or topoisomerase. The nature of primers for LUCA replication would not have been restricted to RNA. In fact DNA primers and RNA primers can be generated equally well by archaeal DNA primase and archaeal DNA replication operates naturally with DNA primers [24], which incidentally eliminates a frequently cited “main support” for the RNA World theory. Complications of lagging strand replication are avoided since each strand would be replicated from its own nick. One single DNA polymerase would replicate both strands to generate complementary single-stranded DNA copies that would anneal readily to form a linear double-stranded DNA. Due to displacement of the nicks in both strands the annealed double-stranded DNA acquires sticky ends (flaps) for circularization. Chromosome length could be seamlessly multiplied by continued rolling circle replication.

The proposed rolling circle replication means that both strands are equivalent carriers of high-density genetic endowments. It explains the universality of ancillary replication factors. A sliding clamp assists the association between DNA polymerase and DNA template. Single-stranded DNA binding proteins protect the emerging single-stranded DNA from premature annealing, from hydrolysis and from forming secondary structures. Flap endonucleases clip excess single-stranded ends of the circularizing DNA replicas.

**LUCA Transcription.** From the homology of genes for the core components of RNA polymerases between the domains Bacteria and Archaea and from their positions in equivalent locations of their consensus gene clusters (Table 1) we project a LUCA RNA polymerase with bacteria-type core components α and β, β’; or α and a common ancestor of β and β’ [25] (Werner theorem). By contrast, the two domains show no commonality in terms of initiation and termination of transcription and corresponding genes are absent from the LUCA consensus gene cluster (Table 1). We explain this by proposing that LUCA practiced rolling circle transcription, proceeding continuously around the plasmid-sized, circular chromosomes, and perhaps multiple times [16,22,23]. Initiation would have occurred (without promoter) at a DNA nick or as seamless transition from rolling circle replication. At the time of transition from precell growth to precell reproduction the process of rolling circle transcription would have been terminated by transition to rolling circle replication. This would have been caused simply by conversion of ribonucleotides into deoxyribonucleotides, catalyzed by a bacterial/archaeal anaerobic class III reductase, whose [4Fe4S] reaction center bears a relationship to the FeS-World theory [26] (Reichard theorem). Like extant archaeal primase [24] LUCA RNA polymerase would have operated equally well with ribonucleotides and deoxyribonucleotides. Instead of controls of transcription of individual genes, rolling circle transcription would have automatically ensured properly timed expression of genes by the order of their occurrence in the genome, which explains gene order conservation in the domains Bacteria and Archaea (Table 1).

**LUCA Translation.** As evidenced by the LUCA consensus gene cluster (Table 1) LUCA must have been in possession of a rather modern ribosomal machinery; and the presence of all genes for the ribosome-polymerase tether (S10--NusG--Rpo β) indicates that LUCA translation must have occurred in tandem with LUCA transcription. This tether prevented premature abortion of transcription, adjusted the rate of transcription to that of translation [27,28] (Nudler-Rösch theorem) and obviated LUCA translation initiation factors for individual genes. Indeed, the LUCA consensus gene cluster (Table 1) is devoid of genes for translation initiation factors. Translation merely had to begin by leaderless initiation and then proceed in sync with the movement of the mRNA out of the transcription machine. Some leaderless initiation is operative to this day in all three domains. It seems to be primitive, since it is insensitive to cross-domain combinations of mRNAs and ribosomes, and it may have proceeded in LUCA with undissociated, yet otherwise complex ribosomes [29] (Londei theorem).

The elongation factors EF-Tu(EF1α), EF-G(EF2) in Bacteria (and Archaea), whose genes are present in the LUCA consensus gene cluster (Table 1), are projected back to LUCA and must have diverged from a common precursor elongation factor that also served as auxiliary factor for peptide release (as EF1α in extant Archaea) by ester hydrolysis. This system for termination requires enthalpic catalysis. It is projected back to LUCA and beyond as having always been facilitated by a proteinaceous catalyst. After exiting from LUCA ribosomes mRNAs had to be quickly broken down for enabling transcriptional control. The propensity of mRNAs to self-destruct intramolecularly due to 2’-OH is therefore a built-in virtue rather than a vice. Finally, LUCA had to be in possession of means for the hydrolysis of useless proteins in order to prevent the closed precells from becoming clogged.

**Unitary LUCA Genetics.** Telling by the size and complexity of the genes in the LUCA consensus gene cluster and by their conserved sequences, LUCA must have had a complex genetic machinery with four canonical bases coding for the canonical set of amino acids. Correpondingly, LUCA must have had an extensive metabolism for monomer synthesis, nutrient assimilation, bioenergy transformations, lipid synthesis, coenzyme synthesis, salvage pathways and catabolic capabilities. This enormous complexity of LUCA means high vulnerability to replication errors. Therefore, error correction mechanisms, extant types or simpler precursors thereof [30,31], must have been active in LUCA genetics. Remarkably, the long patch base excision repair mechanism is reminiscent of rolling circle replication as evidenced by the fact that several ancillary replication factors (e.g., sliding clamp, 5’ single-stranded flap endonuclease) are also operative in the repair machinery. This completes our picture of a unitary character of LUCA genetics: a coherent rolling circle system for replication, transcription, translation and long patch base excision repair. We may even speculate that nicks for initiating replication were identical with sites of base excision for damage repair.

Transcription and translation are interdependent, one generating the catalysts for the other. They are also serially connected and their machineries are even physically tethered. In this overall scheme of backward convergence towards a unitary machinery we may suspect the invisible hand of chemistry at work. Indeed, the severe restrictions that are inherent in the laws of chemistry may well be the ultimate cause for continued chemical simplicity, consistency and coherence over billions of years of post-LUCA evolution.

## 3. Thermal Course of Evolution

The extant biosphere is spread over a wide range of growth temperatures, which poses a crucial question [32]: What is the direction of evolution of the optimum growth temperature of organisms (in short “thermal evolution”)—Is it reversible or irreversible? Phylogenetic considerations suggest a hyperthermophilic origin, but they are inconclusive from a formal point of view. Chemical considerations, however, offer a definitive answer to our question:

(1) Thermally upward evolution (e.g., from mesophiles to hyperthermophiles) would require mutations for all thermally relevant features, notably for increasing the stability of all folding structures. Now, any such mutation has selective value in a thermally challenging environment, if and only if it strengthens the thermally most challenged feature, *i.e.*, the thermally least stable structure. This means that all features would have to adapt thermally upwards in a specific order. Moreover, all thermal adaptations of whole organisms are restricted to small temperature increments, as evidenced by the typical thermal growth curve of bacteria with a gradual rise from the minimum growth temperature to optimum growth temperature and a steep drop between the optimum growth temperature and the maximum growth temperature. Thus, thermally upward adaptation of whole organisms could occur only at an extremely low rate, which renders it probabilistically forbidden. Thermally downward evolution by contrast proceeds by haphazard losses of thermal features, notably structural stabilizers, which do not have to occur in a specific order, nor by tiny increments.
(2) Some protein folding structures are stabilized by sets of ligators (e.g., pairs of C-X-X-C) that form strong coordination bonds to transition metal centers (e.g., Fe^2+^, Zn^2+^). For thermally upward adaptation by the emergence of such cross-linking the set of ligators would have to be installed simultaneously by sequence covariation, which is improbable. In thermally downward evolution, however, losses of ligators may occur haphazardly, one-by-one. This explains why some proteins (e.g., aminoacyl synthetases and ribosomal proteins) show on average a decrease, but never an increase, of the number of C-X-X-C signatures in the direction from hyperthermophiles to psychrophiles [33,34]. Such signatures are sometimes maintained in mesophiles as molecular relics or, when lost recently, e.g., by single cysteine removal, they may reappear as molecular atavisms.
(3) Kinetic experiments show that in the presence of an enzyme a biochemical reaction requiring enthalpic catalysis shows typically a gentle drop of reaction rate with decreasing temperature. When such reaction is carried out in the absence of an enzyme the rate decrease with decreasing temperature is extremely steep, and steepness correlates with the sluggishness of the reaction. At the temperature of mesophiles the non-catalyzed reaction rate is so extremely low that early catalysts with low catalytic activity could not have increased the reaction rate to a metabolically useful level [35,36]. The conclusion is inescapable for multi-step metabolic pathways: Only at temperatures typical for hyperthermophiles (or above) would rates of all involved uncatalyzed reactions be high enough to be readily augmented to metabolically required rates by modest catalytic innovations. Thus, the evolution of catalysts for metabolic pathways could only have started at high temperatures, and subsequent thermally downward evolution of enzymatic catalysts would automatically generate metabolic rates much higher by many orders of magnitude than corresponding rates in a mesophilic origin of life. The fact that some uncatalyzed reactions are relatively fast at low temperatures is here of no concern, because a pathway of reactions is as slow as its slowest step. The hyperthermophilic requirement of early life must have persisted as long as the catalytic activities of the evolving catalysts remained relatively low. In any event, all biochemical reactions, whose non-enzymatic parent reactions face a very high catalytic burden, must have entered the metabolism in the all-hyperthermophilic phase of early life. An evolution to lower and lower temperatures became possible to the extent that catalysts evolved to acquire higher and higher catalytic activity, and this evolution was possible, because the initial catalytic burden was not too high. With the appearance of the genetic machinery essentially all metabolic reactions came under enzymatic control and this enabled an evolution to mesophilic and psychrophilic lifestyles. Thermal rate leveling at the origin of life was thus replaced by adaptational rate leveling (Wolfenden theorem I).
(4) From high temperature at the origin of life evolution would have proceeded by successive metabolic expansions and by migration to regions of lower temperature with compensation of lower reaction rates by more effective catalysts. Peptide/protein folding would have originated with a few locally focused cross-links due to strong, covalent coordination bonds to transition metals in an otherwise flexible folding structure. In the course of early evolution this crosslinking by a few, focused coordination bonds would have been first supplemented and later substituted by a multitude of weak, non-covalent group interactions. This could only have occurred after translation had reached the required accuracy. With decreasing temperature the functionally feasible sequence space for peptides and polynucleotides expanded. In addition to this thermally vertical (downward) evolution there would have been a thermally horizontal (isothermal) evolution, increasing the functionally feasible sequence space by secondary stabilizations, e.g., by methylation of RNAs at strategic 2’-positions to prevent self-cleavage.

## 4. Place of RNA in the Evolving Mechanism of Evolution

**Figure 2:**
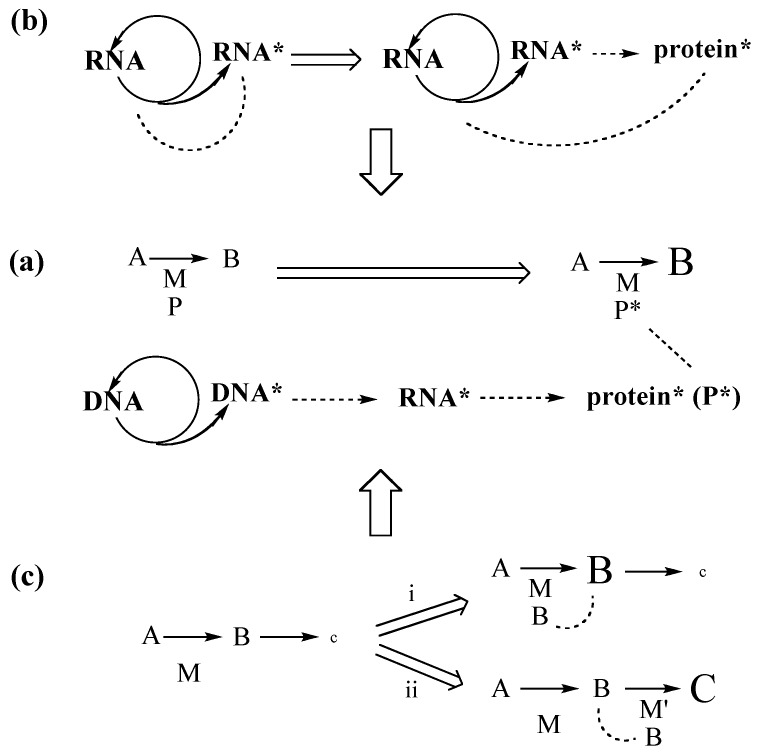
Two views of evolving evolution (open double arrow: evolutionary change; closed double arrow: evolution of evolution).

Extant organisms evolve indirectly (Figure 2a). Replication of DNA generates variant DNA*, which is transcribed into variant mRNAs*, which in turn is translated into variant proteins (P*). These become ligands of variant metallo-enzymes (MP*) (non-metallo-enzymes are here ignored), which boost a metabolic reaction (A → B). This indirectness is the essence of extant genotype-phenotype dichotomy.

According to the **RNA World theory** [37] life began with a population of randomly replicating, “living” RNA molecules (perhaps inside lipid vesicles) within a cold “prebiotic broth”. Varied RNA* molecules were feeding back into the replication cycle (Figure 2b). Within this RNA replication context a process of translation emerged that converted a medley of amino acids into an undefined library of peptides/proteins. Some of these are expected to somehow have fed back into the replication/translation system. Neither transition metal catalysts nor metabolic redox reactions have a place in the picture. Nomenclature within this theory distinguishes between “prebiotic” chemistry or chemical “evolution” before the onset of replication and “genetic evolution” after the onset of replication.

The RNA World theory is faced with insurmountable obstacles. By one account [38] the origin of translation in a prebiotic broth requires a set of at least 13 functional RNA molecules with a total of at least 1800 nucleotides. Its chance appearance has an extremely low probability of less than 10^−1018^—effectively zero over the available space and time of the Universe. Therefore, the author concludes that our Universe must be part of an eternal Multiverse with an infinite number of universes. According to another RNA World calculation the lengths of RNA genes must remain below an abrupt threshold, for otherwise their replication would lead inevitably to an error catastrophe [39,40]. This predicament is further aggravated by the fact that protein folding by weak group interactions requires a minimum folding-appropriate length of the protein chain. While there is criticism (not rebutted so far) that the threshold abruptness is dependent on arbitrary, unrealistic assumptions [41], the error catastrophe proposal certainly is compatible with the conclusion that a spontaneous origin of translation in the RNA World context would have been an improbable affair.

According to the **FeS World theory** [22,23,33,42,43] life began in a hot volcanic-hydrothermal fluid flow with formation of low-molecular-weight organic compounds from volcanic-hydrothermal nutrients, driven by local chemical energy and facilitated by catalytic transition metal centers of contacted crustal minerals. In this mineral context iron sulfide is today and has always been omnipresent and multi-functional. Catalytic nickel centers by contrast are today restricted to a few biochemical niches, but may have been most crucial at the beginning [44]. Sources for nutrients originate by magmatic/crustal mineral reactions, notably with graphite and N_2_: e.g., CaCO_3_ → CaO → CaC_2_ → CaCN_2_ → Ca(CN)_2_ or CO_2_ ↔ CO ↔ C^0^. Hydrolysis at hydrothermal sites and/or quenching provides super-equilibrium nutrient activities. From this vantage point evolution began with a direct metabolic mechanism (Figure 2b) [22,23], which is related to the chemical concept of ligand-accelerated catalysis [45]. According to this mechanism an organic product B of a synthetic reaction turns directly into a ligand of a transition metal catalyst (M) with the effect of increased catalytic activity. Two lines of evolution must be distinguished: (i) product B turns into a ligand of transition metal catalyst M for the synthesis of B (autocatalytic feedback effect); (ii) product B turns into a ligand of transition metal catalyst M’ for the synthesis of another product C (feed-forward effect). From this primordial mechanism of evolution the extant mechanism of evolution evolved by a multitude of incremental transformations without any essential discontinuity. Nomenclature within the FeS world theory distinguishes between “abiotic chemistry” and “biotic chemistry” and applies the name “pioneer organism” to a chemical system of ligand feedback and feed-forward effects that initiated a directional series of transformations from the intrinsic finality of inanimate chemical reactions to the unending quest of biological evolution.

We shall now flesh out these two competing general courses of evolution by more and more detailed accounts. We begin with proteins, raison d’être of the extant genetic machinery.

## 5. From Primordial Peptide Cycle to Genetic Protein Cycle

The sequences of large protein molecules comprise several distinct contiguous sequence segments that fold autonomously into distinct “structural domains”. These are hinged together to collapse into an overall folding structure [46]. Short protein molecules form “single-domain” folding structures. This pattern suggests that early on the process of translation generated short protein molecules with single-domain folding structures and that only later in evolution were long oligo-domain proteins formed by gene fusion, which greatly simplifies the problem of early protein evolution (Rose theorem). Before we address it we should clear up a possible terminological confusion. The chemical term “peptide” covers a vast class of heterochiral compounds from dipeptides to macromolecular polypeptides—homopolymers as well as copolymers. The biological term “protein” defines natural, genetically encoded, homochiral polymers of amino acids. The discrepancy between polypeptide irregularity and protein regularity looses some of its force once we realize that primordial peptides could have been formed from a small set of amino acids by (heterogeneous or homogeneous) transition metal catalysts of great selectivity (akin to syndiotactic or isotactic polymerization, or regioselective copolymerization). With this in mind we now ask: How can we account for the origin of peptides and for the transition to folded proteins?

**Metallo-Peptides.** In the context of the FeS World theory amino acids are formed by carbon fixation with transition metal catalysis [47,48,49]. These pathways have intrinsic chemical restrictions. The set of autotrophically generated amino acids is therefore quite limited and a chaotic mélange is avoided from the start. Moreover, the problem of a minimum length threshold for functional, folded proteins disappears, because very short oligopeptides (or even amino acids) can turn into ligands of transition metal catalysts. In addition, for ligand-accelerated catalysis heterochirality of ligands is a benefit rather than a detriment [22,23,50]. Any pre-ribosome would then have had the modest burden of generating the same kind of short peptide ligands that were already part of the metabolism. Thus the FeS World theory replaces the intractable problem of an abrupt origin of autonomously folded protein catalysts by the simpler problem of the biosynthesis of short peptide ligands for metallo-peptide catalysts. From this vantage point the formation of folded proteins is not such a big step. It merely means a transition of the peptide-metal relation from that of a short peptide ligand in the ligand sphere of a metal center to that of a catalytic metal center inside a larger folded peptide ligand. In this transition by a series of small incremental changes coordination bonding is maintained as an invariant structural element. Moreover, for thermo-stable folding of a peptide with a few focused crosslinks by coordination to transition metal centers the peptide sequence requirements are quite relaxed. By contrast, peptide folding by a multitude of distributed, weak group interactions is highly sensitive to sequence variations. It requires high-fidelity information transfer and could have come about only relatively late in evolution. Thus, the appreciation of transition metal catalysis and crosslinking allows us to explain the origin of translation by a stepwise progression of ligand synthesis without any bottleneck of improbability.

**Protein Cycle.** At the chemical core of extant genetics lies an energy-coupled protein cycle. It requires energy coupling with ATP hydrolysis and all its steps require enzyme catalysis. Amino acids are converted via aminoacyl-AMPs (aa-AMPs) (anhydride energy) into aminoacyl-tRNAs (ester energy), from there into proteins (amide energy) and finally back to free amino acids. The formation of aa-AMP and subsequent aminoacylation of tRNA require enthalpic catalysis by transition state stabilization as well as channeling of the hydrolytically sensitive aminoacyl-AMP intermediate. Formation of aa-AMP presents an extremely high catalytic burden [51], which increases drastically with decreasing temperature. Enzymes with their diversity of amino acid units and high conformational flexibility are ideally suited for enthalpic catalysis by stabilizing the transition state, while relative lack of chemical diversity and flexibility of RNA militate against transition state stabilization by ribozymes. By contrast, ribosomal catalysis of peptide bond formation is mainly entropic [35,36] (Wolfenden theorem II). It operates by restricting freedom of movement, positioning and orientation of the reacting moieties (scaffolding), and by exclusion of water from the reaction center, thus lessening the entropic cost of organizing water molecules around the transition state. The burden of this catalysis does not change with temperature. Bonding of tRNA to rRNA is ideally suited for this purpose. We are thus faced with a clear division of catalytic labor between the predominantly enthalpic catalysis of amino acid activation and aminoacylation of tRNAs by aminoacyl-tRNA synthetases and the predominantly entropic catalysis of peptide bond formation by the ribosomal machinery. This fundamental chemical division of labor must have been in play at, and even before the dawn of ribosomal translation: Let us see how this pattern traces back in time towards the origin of life.

**Figure 3:**
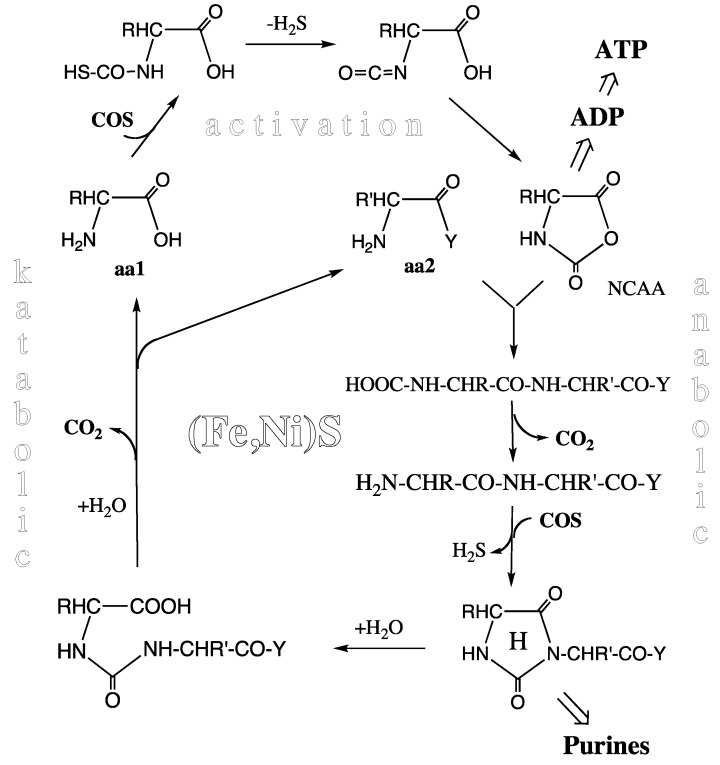
Peptide cycle (aa1, aa2 = amino acids; Y = –OH or –NH-CHR-CO-Y; R, R’ = amino acid side chains; H = hydantoin ring; NCAA = N-carboxy-amino acid anhydride).

**Peptide Cycle.** At or near the level of the pioneer organism we see an experimentally demonstrated unitary peptide cycle, or better a concatenation of peptide cycles (Figure 3) each composed of a synthetic (anabolic) segment with N-terminal elongation and a degradative (katabolic) segment with N-terminal shortening, both segments driven and catalyzed by essentially the same mechanism [52]. In the activation step COS (derived from CO and H_2_S by Ni-catalysis) reacts with amino acids to form highly energetic N-carboxy-aminoacyl anhydrides (NCAAs) via thiocarbamates. This activation reaction requires enthalpic catalysis, but compared to activation with ATP it has a lower catalytic burden due to the neutrality of the COS molecule. In the synthetic step the NCAAs react with a free amino group of another amino acid or of a peptide. The entire reaction sequence may be catalyzed by a mineral surface. Catalysis by a pyrite surface with sulfur deficiencies has been demonstrated [53] (Marx theorem). Mineral catalysis is partly entropic due to two-dimensional positioning and orientation of the reactants and due to lowering of the water activity.

**Ribonucleosyl Phosphates and Coenzymes.** As a windfall profit of the peptide cycle, the NCAAs could have provided energy coupling, perhaps even by reaction with phosphate to form aminoacyl phosphates (AAPs), which subsequently could react serially with ribonucleosides (N) to form the all-important ribonucleosyl (mono/di/tri)phosphates of increasing energy content: N + AAP → NMP; NMP + AAP → NDP; NDP + AAP + NTP [22]. Ribonucleosyl phosphates may also form by volcanic-hydrothermal reaction via acetyl-thioester [54] and acetyl-phosphate [55]. The ribonucleosyl phosphates were available not only for the formation of oligoribonucleotides or RNA, but also for activating other metabolites so that they could undergo reactions that would otherwise be endergonic, such as post-translational modifications of proteins. Instructive here is the case of activation of coenzymes (erroneously counted as support for the RNA World theory). Phosphopantetheine is activated with ATP to form coenzyme A with a diphosphate bridge. Coenzyme A then reacts with the apoprotein of an enzyme to covalently attach phosphopantetheine. Such covalent attachment of a cofactor to an enzyme means a lower dependence on high sequence fidelity than the non-covalent mobile attachment of coenzyme A, which is seen as a latecomer. ATP-activation for covalent attachment is also known for lipoate and biotin. Similar covalent coenzyme anchoring, may have once existed for FAD, NAD and molybo/tungsto-pterins, but has not (yet) been found in extant biochemical systems [43]. Incidentally, activation of pyruvate would have given rise to N-terminal pyruvoyl-peptides as functional precursors of pyridoxal phosphate [43].

**Figure 4:**
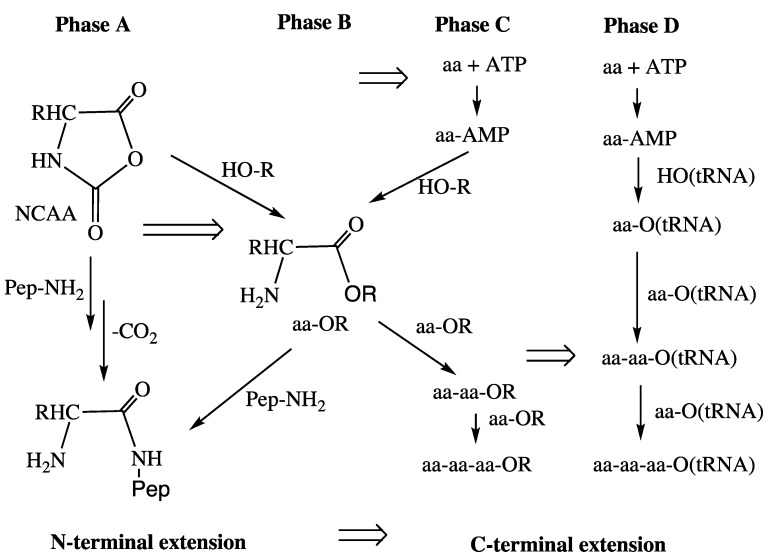
Evolution of peptide synthesis (Pep-NH_2_ = peptide with free amino group).

**From Peptide Cycle to Protein Cycle.** Evolution from the primordial peptide cycle to the LUCA protein cycle may be sketched formally as a transition (Figure 4) with the following four phases. Phase A: N-terminal extension of peptides by NCAAs; Phase B: formation of ester-activated amino acids (aa-OR) by reaction of NCAAs with a primitive tRNA precursor (HO-R), e.g., an RNA microhelix, to form an aminoacyl ester and subsequent N-terminal peptide chain extension; Phase C: C-terminal extension of an ester-activated amino acid, aa-OR (or ester-activated peptide) by reaction with an ester-activated amino acid (aa-OR), derived from NCAA or aa-AMP; Phase D: extant conversion of an amino acid by ATP via aa-AMP to aa-O(tRNA). Peptide products of all phases would feed back into the amino acid activation or aminoacyl transfer reaction by operating as primitive aminoacyl-tRNA synthetases. In the course of these transformations the peptide synthesis mechanism changes from mineral surface catalysis to ribosome surface catalysis, *i.e.*, from partly entropic to fully entropic catalysis. In phase C aa-AMP would be formed from ATP and used immediately without entering an aqueous pool.

The transition to ribosomal peptide bond formation with charged tRNAs is a transition from N-terminal peptide elongation to C-terminal peptide elongation. In the transition phase both elongation types must have coexisted. This means that for some time N-terminal peptide cleavage by COS coexisted with C-terminal protein elongation by the ribosome. As a consequence C-terminal protein elongation by the ribosome would have been countermanded by N-terminal peptide cleavage. It is attractive to speculate that the initiation of translation by N-formyl-Met-tRNA_i_ served originally the purpose of preventing N-terminal peptide cleavage and was later preserved as molecular relic. Evolution from highly energetic NCAAs (anhydrides) to weakly energetic aminoacyl-tRNAs (esters) means increase of selectivity, enabling increase of complexity and functional diversity.

## 6. Place of RNA in the Origin and Early Evolution of Translation

Our sketch of the transition from peptide cycle to protein cycle has led us to the threshold of the translation machinery. We now try to retrace major strands of its origin and early evolution and begin with the ribosome.

**Origin and Evolution of the Ribosome.** Extant biosynthesis of ribosomes [56] provides us with insights into ribosome evolution. In the domains Bacteria and Archaea it begins with the transcription of the rRNA operon to form a primary transcript: --16SrRNA--tRNAs--23SrRNA--5SrRNA--tRNAs--, whereby the symbol “--” represents transcribed external and internal spacer sequences. As the primary rRNA transcript emerges from the transcription machine, it acquires rRNA folding structures, while it is being coordinated to ribosomal proteins and to Mg^2+^ ions, and while some nucleotide modifications are also occurring at that stage. Subsequently the resulting pre-ribosome is cleaved into the two subunits, which then are completed separately. To this biosynthesis we apply the Florkin-Granick rule [57,58,59]: the order of steps in a biosynthetic pathway recapitulates the order of their emergence in evolution. By this rule the early ribosome had a unitary un-dissociable structure akin to the extant pre-ribosome [42]. Subsequently the unitary ribosome would have evolved into a two-subunit ribosome (with LSU and SSU) that retained its undissociated form. We find an echo of this evolutionary stage in extant leaderless translation by dissociable, but un-dissociated ribosomes [29]. It follows from this hypothesis that the extant ribosome as mRNA-programmable catalyst is the result of a long coevolution of the LSU and the SSU. The coevolution must have begun with the formation of an ur-ribosome by the fusion of an ur-LSU as a minimal peptidyl transferase center (PTC) and an ur-SSU as a minimal decoding unit. This fusion would have facilitated the formation of intersubunit bridges as intramolecular folding determinants rather than intermolecular association determinants. Where did the ur-LSU and the ur-SSU come from? According to a novel cladistic heuristic, applied separately to rRNAs and r-proteins of whole ribosomes (LSU + SSU), the ur-SSU and ur-LSU result from two separate deep pre-ribosomal tracks of evolution [60] (Caetano-Anollés theorem I). The functional precursors of the ur-SSU may have functioned as a primitive RNA polymerase, *cf.* [61], while the functional precursors of the ur-LSU may have functioned as catalysts for forming peptides without mRNA programing.

Ribosomal coevolution has now become tractable by combining knowledge of crystal structures of extant ribosomes with considerations of the logic of the chemical situation [61]. The most detailed conclusions have been reached for the LSU. Its globular r-proteins are distributed over the surface with long protein extensions filling deep cracks between rRNA segments like mortar [62]. It was further found that the PTC consists of a small contiguous segment of 165 nucleotides with a two-fold pseudo-symmetry, which is surmised to have emerged by duplication of a segment of 82 nucleotides. It folds to form two templates for binding the 3’-terminal CCA segments of two tRNAs in proper spacing and orientation for peptide formation by ester aminolysis [63,64] (Yonath theorem). Moreover, the LSU was found to have expanded radially from the PTC by the addition of terminal unpaired rRNA extensions that folded onto peripheral RNA double helices without disturbing the integrity of the pre-established core [65] (Bokov-Steinberg theorem). In the LSU of the domains Bacteria and Archaea 4 RNA-crosslinking dimagnesium tetraphosphate microclusters have been located in the immediate vicinity of the PTC [66]. Their evolutionary context is revealed by a cleverly simplified, yet astonishingly informative LSU growth model. The 3D structure of the LUCA LSU (with spherical coordinates) was obtained by superposition of the 3D structures of a bacterial LSU and an archaeal LSU and subdivided into spherical growth shells with the PTC in the center. Looking in radial direction and thus along the arrow of time, we see a decrease of density of crosslinking Mg^2+^ ions. It is compensated by increases of RNA base pairing and of r-protein density and folding [67] (Williams theorem). The Mg^2+^ ions may have been preceded by crosslinking Fe^2+^ ions [68]. Such a crosslinking Mg-cluster has also been located in the SSU [69], which speaks for its antiquity *cf.* [60]. These results support the theory that in the course of early evolution strong metal-phosphate crosslinkings as structural determinants were replaced by weak group interactions. Finally, convincing arguments have been presented that the changeover to the homochirality of life, *i.e.*, the transition from a mere enantiomeric excess to the extant homochiralities of nucleic acids and proteins, must have occurred in the early phase of ribosomal evolution [61] (Fox theorem).

In view of the absence of peptide components from its interior the extant PTC is widely surmised to be a ribozymic vestige from (and evidence for) a primordial “RNA World”, e.g., [63,70]. There are problems with this line of reasoning. (1) Catalysis by the PTC is entropic [35,36]. Therefore, the laws of chemistry rather than historicity dictate the presence of entropy-catalytic rRNA and the absence of peptide interference in the reaction center. Thus, the vestige reasoning seems to be a *non sequitur*; (2) It should be stressed that entropic catalysis requires stability of location and orientation of RNA sequences and it is not unrealistic to assume that peptide extensions of r-proteins, which reach just to the periphery of the PTC (not into the very catalytic center) may well be mandatory for providing the required stability of the larger conformational context of the PTC scaffold without interfering with its entropic catalysis, *cf.* [71]. This consideration is consistent with the proposed evolutionary direction from enthalpic to entropic catalysis (Figure 4); (3) Moreover, the presence of crosslinking Mg-clusters seems to speak against an autonomously folded PTC reaction center. To sum up, the PTC is today and may always have been, at least since the inception of the ur-ribosome, a metallo-ribonucleo-peptide particle and surely not a mere ribozyme.

**tRNA Evolution.** Extant tRNAs may be viewed as mobile components of the ribosome. Their 3’-terminal ACC segments are scaffolded by the PTC for entropic catalysis and this relationship goes back to the ur-ribosome. Crucial post-transcriptional modifications of tRNAs with the effect of improved specificity and thermostability [72] are also projected far back to pre-LUCA times (McCloskey theorem). Many extant tRNAs are transcribed from genes that do not code for the catalytically crucial 3’-terminal CCA segment. Rather, this segment is post-transcriptionally added by CCA-adding enzymes with serial utilization of CTP and ATP [73]. These enzymes show little cross-domain homology, which indicates late, independent emergence. Thus, the earliest tRNA genes must have coded for whole tRNAs including their CCA segments and we still find some genes for whole tRNAs in each of the three domains of life. The CCA segment is today (and must have always been) vulnerable for cytosine deamination. This suggests that before the time of LUCA damaged CCA segments were repaired by a repair enzyme that later diverged into the different CCA-adding enzymes in the Bacteria and Archaea and these enzymes then provided cover for the polyphyletic spreading of tRNA genes devoid of CCA-coding.

The tRNAs seem to have evolved from short hairpin precursors with a terminal unpaired sequence (e.g., CCA) [74] (Schimmel theorem I) or even from a precursor in the form of a sole unpaired sequence (e.g., CCA) [75] (Steitz theorem). They must have coevolved with the ribosome to reach an early structural completion in a master tRNA (cloverleaf secondary structure, l-shaped tertiary structure) during the infancy of the ribosomal translation machinery with subsequent divergence into the set of extant tRNAs, *cf.* [76]. The topological course of this evolution to the master tRNA structure is subject to diverse proposals, e.g., [61,77,78,79].

**Aminoacyl-tRNA Synthetase Evolution.** The aminoacyl-tRNA synthetases form the bridge between metabolism and genetics. For explaining this connection we proceed in the retrodictive direction. The first clue comes from a comparison of the 3D structures of aminoacyl-tRNA synthetases. It reveals a fundamental division of these enzymes into two classes, each for about 10 amino acids [80,81]. Class I enzymes bind to the minor groove of the tRNA acceptor arm and charge the terminal 2’-hydroxyl group, while class II enzymes bind to the major groove and charge the terminal 3’-hydroxyl group [82]. The acceptor arm (Acc) of tRNAs can bind simultaneously to a class I synthetase on one side and to a (presumably ancient) catalytic core domain of a class II synthetase on the other side, or *vice versa* [83]. Remarkably, simultaneous synthetase binding obeys subclass pairing (Ia-tRNA-IIa; Ib-tRNA-IIb; Ic-tRNA-IIc), while cross-subclass pairings appear to be sterically inhibited. The finding has led to an evolutionary proposal: As starting point a micro/mini-helix as ur-tRNA (~Acc) was bonded to an ur-class I synthetase (at minor groove) and also to an ur-class II synthetase (at major groove) with the benefit of stabilization of the RNA helix against thermal degradation. This archaic ternary complex (urI-Acc-urII) evolved divergently into three ternary complexes (urIa-tRNA-urIIa; urIb-tRNA-urIIb; urIc-tRNA-urIIc) and these in turn gave rise to the extant aminoacyl-tRNA synthetases with conservation of the classI/classII relationships (Schimmel theorem II):
Ia: Val-Ile-Leu-Met-Arg-Cys Ib: Glu-Gln-Lys Ic: Tyr-TrpIIa: Ala-Ser-Gly-Thr-Pro-His IIb: Asp-Asn-Lys IIc: Phe-Pyl-Sep


A daring theory [84] suggests that early on class I and class II aminoacyl-tRNA synthetases were coded opposite one another on the same ancestral gene, one on the (+)-strand and the other in the reverse direction on the (−)-strand, and with their reading frames in register (in-frame sense-antisense coding). Indeed, short, class-defining sequence motifs of the catalytic centers of enzymes of both classes show significant sense-antisense homology; and the most conserved (hence oldest) sequence motif of one class comprises predominantly amino acids that are activated by the synthetases of the opposite class (Rodin theorem). Incidentally, sense-antisense coding has also been retrodicted for tRNAs [85] and it was found to still exist in extant genomes [86]. It was found that class I Trp-tRNA synthetase and class II His-tRNA synthetase still bear the mark of ancestral sense-antisense coding. More importantly, it was possible to retrodict and reconstruct ancient, small, invariant catalytic cores (120–130 residues) of these two synthetase classes. Experiments with these peptides, termed “urzymes”, showed that both interact with tRNA acceptor arms (Acc) and that both have the same (essentially modern) catalytic profile in terms of high activities (~60% of extant enzymes) for amino acid activation with ATP and tRNA acylation, and in terms of rate leveling between activation and acylation. They may well go back to an early phase of the evolution of RNA-guided peptide synthesis with a small repertoire of amino acids: Class I for nonpolar and class II for polar amino acids, as required for molten globular structures. Moreover, the modul structures of these urzymes have been interpreted as pointing to two still deeper and simpler catalytic peptides of both classes with sense-antisense coding, which may well have had already the function of directing the synthesis of nonrandom peptides [87] (Carter theorem).

It has been deduced that primordial translation must have faced a genome with systematic in-frame sense-antisense triplet coding, whereby the informationally crucial central base in each triplet was flanked by two outside bases that were relaxed in their significance, thus making best use of the memory molecules at an early time when chromosomes were inflicted with intrinsic length-restriction [88] (Rodin-Carter theorem). At and before the level of LUCA this proposal fits nicely to the proposal of a rolling circle expression of both (equivalent) DNA strands with rigid gene order. It strengthens the notion of a backward convergence toward a unitary origin of the genetic machinery. Subsequent evolution proceeded in the direction of ever-longer chromosomes. Sense-antisense coding (highly restricted for two proteins and more relaxed, if one strand codes for an RNA operon) was later replaced by sense-only coding, whereby the original equivalency of both strands was broken and freedom of information coding increased [88].

The question of the origin of the aminoacyl-tRNA synthetases leads to a conceptual impasse. The synthetases are proteins required for the ribosomal synthesis of proteins. Within the RNA World theory this impasse is overcome by the assumption that the aminoacyl-tRNA synthetases were at first ribozymes and that they were replaced by enzymes after the appearance of a sufficiently advanced ribosomal translation system. According to the FeS World theory the impasse is overcome by the assumption of a coevolution from monomers (amino acids and nucleotides as metallo-ligands) via oligomers (metallo-peptides and metallo-oligonucleotides) to polymers (metallo-proteins and metallo-RNAs). Let us see how these two alternatives fare with the chemical fundamentals, notably with the hydrolytic instability of aminoacyl-AMP (aa-AMP); and with the clash between mainly enthalpic catalysis of aminoacyl-tRNA synthetases (amino acid activation and aminoacyl transfer to tRNA) and entropic catalysis of ribosomes and ribozymes.

Considering first the RNA World theory, the key intermediate in the charging of tRNAs, aa-AMP, is extremely sensitive to hydrolysis under neutral conditions [89] due to the presence of a free α-amino group, and increasingly so with increasing temperature. This is the reason why aminoacyl-tRNA synthetases produce aa-AMP as intermediate that is immediately channeled to react with tRNA without entering an aqueous pool. In the context of the RNA World theory aminoacyl-tRNA synthesis should have always operated with ATP and with immediate internal channeling of aa-AMPs for reaction with tRNA. They would have had this binary catalytic function first as ribozymes and later as enzymes. It is difficult to see how such a binary ribozymal catalyst could have come about in the first place and how it could be implemented in the laboratory. Therefore, attempts to demonstrate experimentally the possibility of a ribozymal aminoacyl-tRNA synthetase have focused typically on separately optimized ribozymal reactions for amino acid activation and aminoacyl transfer. For example, in a first reaction an amino acid was activated to aa-AMP at ~pH4, thereby eliminating the deleterious free amino group of aa-AMP by protonation [90], while in a second, separate reaction system aa-AMP was reacted as bulk starting material with RNA to form aa-RNA, which then reacts with more aa-AMP to form aa-aa-RNA, whereby ~pH7 was chosen to ensure the required free amino group of aa-AMP and the temperature was lowered to the unrealistic value of 0 °C so that the desired synthetic reaction could outcompete aa-AMP hydrolysis [91]. In other ribozyme experiments rapid self-destruction of the activated amino acid has been avoided by employing amino acid activation in the form of an artificial cyanomethyl ester [92], or by activating an amino acid with a 5’-trisphosphoryl-RNA instead of ATP [93]. In these strategies entropic catalysis [35,36] involving two or more ribozymes (RNAs) seems to work well, while enthalpic catalysis of amino acid activation with ribozymes for amino acid activation seems to be modest.

According to the FeS World theory (Figure 4) activation of amino acids would have begun as formation of NCAAs with a low enthalpic burden to be later replaced by activation with ATP with a high enthalpic burden. By the same token, peptide formation would have been transformed from N-terminal extension to C-terminal extension, whereby the catalytic burden changed from mainly enthalpic to mainly entropic. Since facile formation of peptides via NCAAs has been demonstrated experimentally, this evolutionary scheme is less problematic than the one according to the RNA World theory.

Chemical selectivity (fidelity) of the process of translation resides primarily in the ability of aminoacyl-tRNA synthetases to distinguish between different side groups of the amino acids. Some of these are chemically rather non-distinct and highly similar to others: e.g., Ser/Thr, Phe/Tyr, Ala/Gly, Ala/Ser, Val/Ile, Val-Thr [94]. This similarity leads to misacylations, which are corrected by editing domains of the aminoacyl-tRNA synthetases. Some of these editing features must have come into existence long before LUCA. In the earliest phases of the evolution of translation, however, no editing capability could have existed. According to the RNA World theory of an origin of life in a prebiotic broth with a chaos of amino acids the earliest translation machinery is said to have generated “statistical proteins”, wherein amino acids with similar charging characteristics would randomly replace each other, so that no two protein molecules were likely to have had the same amino acid sequence [94]. Within the context of an autotrophic origin by carbon fixation in a surface metabolism according to the FeS World theory we have to rethink this problem. For this purpose we shall now broaden our evolutionary perspective of translation to include the biosynthesis of amino acids. We shall find that the FeS World theory precludes a chaos of amino acids and by extension a chaos of peptides or proteins.

**Evolution of Amino Acid Biosyntheses.** At the earliest stages of evolution according to the FeS World theory amino acids are metabolic “end” products in the sense that they turned into ligands for modulating catalytic transition metal centers, while later the amino acids became intermediates in pathways for the non-ribosomal synthesis of peptides and still later for the ribosomal synthesis of peptides/proteins [22,23,33,50]. A close metabolic connection between amino acid synthesis and ribosomal peptide/protein synthesis has been expressed in the coevolution theory [95] (Wong theorem I). According to this theory translation and the genetic code coevolved in concert with the evolution of biosynthetic pathways to the amino acids and the infiltration of new amino acids into the process of translation. Actually, this infiltration may have occurred by pairs of amino acids with complementary codons [88].

First let us focus on the entry of the α-amino group into the carbon skeletons of the amino acids. Ammonia (NH_3_) or hydrogen cyanide are geochemically unproblematic sources for this purpose. From the premises of the FeS World theory we conclude that the biosynthesis of simple amino acids (e.g., Gly, Ala, Ser or Asp) operated at the level of the pioneer organism by reductive CO/NH_3_ ligand condensation or reductive cyano ligand condensation that formed the carbon skeleton and the functional groups in concert [47,48,49]. It was followed later by a two-step process of carbon fixation to 2-keto acids (or fumarate) and subsequent reductive amination (or NH_3_ addition). It is used to this day for reductive aminations (pyruvate → Ala; oxaloacetate → Asp; α-keto glutarate → Glu) or for amination of fumarate to Asp. Reductive amination with FeS/H_2_S [96] or Fe(OH)_2_ [97] as reductant and catalyst is generally operative for all α-keto acids. At low NH_3_ activity extant reductive amination has to avoid the NH_3_-pool and requires a two-step energy coupling with ATP hydrolysis and with Gln as NH_3_ shuttle: (1) Glu + NH_3_ + ATP → Gln + AMP + PP; (2) 2-keto acid + Gln + reductant → 2-amino acid + Glu + oxidant. This pathway requires an enzyme with NH_3_-channeling and therefore presupposes a sophisticated form of translation. Most extant amino acid syntheses, however, operate by transamination: (α-amino acid + α-keto acid * ↔ α-keto acid + α-amino acid *). It requires pyridoxal phosphate (PLP) as amino transfer shuttle and could have emerged only after the process of translation was already highly evolved.

Let us now look at two different routes for infiltrating peptide/protein sequences with new amino acids. A long route begins with *de novo* biosynthesis of a free amino acid, which is subsequently activated and charged to a tRNA and then incorporated ribosomally into a peptide/protein, where it finally has a function. This long route is the result of a low-probability evolution, since it implies the co-emergence of three high-precision polymer structures: (1) At least one enzyme for the biosynthesis of the new amino acid; (2) a new aminoacyl-tRNA synthetase; and (3) a new tRNA with a new code assignment. A short route begins with an already established aminoacyl-tRNA, which is then converted to a new aminoacyl*-tRNA by side group modification (aa-tRNA → aa*-tRNA). It can immediately enter the process of ribosomal peptide/protein synthesis. Diversification and codon reservation follow subsequently. In evolution the short route must have preceded the long route (Wong theorem II). For three extant amino acids the short route (Asp-tRNA → Asn-tRNA; Glu-tRNA → Gln-tRNA; phosphoSer-tRNA → Cys-tRNA) coexists with the long route [98] (Söll theorem). For two other extant amino acids only the short route exists (phosphoSer-tRNA → selenoCys-tRNA; Met-tRNA → formylMet-tRNA). The short route has the added advantage that thermally instable free amino acids (e.g., Cys, selenoCys) are avoided.

How would the evolutionary transition from the short route to the long route occur? As a first step a new free amino acid (aa *) would be generated by hydrolysis of aa *-tRNA or of a peptide/protein with an aa *-unit. For reintroducing the free amino acid (aa *) into the process of translation an aa *-tRNA synthetase for charging aa * to a tRNA may derive from the pre-established aa-tRNA synthetase. Finally aa * would be synthesized directly in free form by an amino acid synthetase that modifies the side chain of the precursor amino acid (aa → aa *). It has been demonstrated that the Asn synthetase for the NH_3_-dependent conversion Asp → Asn derived from the Asp-tRNA synthetase [99,100] (Francklyn-Kern theorem).

Post-translational modification is the most direct route for introduction of a new amino acid into a peptide/protein. A prominent example is the conversion of the free amino group of a Lys unit by forming an amide with lipoate or biotin, or by forming a Schiff base with PLP. Another example is the phosphorylation of a Ser or Thr unit. It is here suggested that the Cys unit was first introduced into peptides/proteins by post-translational conversion of a Ser unit with H_2_S, possibly after acylation or phosphorylation. This conversion (R-OH + H_2_S → R-SH + H_2_O) is thermodynamically favorable due to the stability of H_2_O. The next evolutionary stage would have been the short route (phosphoSer-tRNA → Cys-tRNA). The transition from this short route to the long route is still underway in the extant biosphere [101,102]. This proposal explains how cysteine, the amino acid with the most crucial functions (catalyst, ligand, redox, radical, group activation) and with the greatest thermal instability [103], could have entered peptides early, yet coded translation rather late (with only two codons), first by the short route (~3.5 Ga) and then by the long route (~3.0 Ga) [104] (Caetano-Anollés theorem II). Enzymatic persulfide serves as extant sulfur source and *in vitro* it may be replaced by inorganic persulfide (HS_2_^−^) [105], which has a striking relationship to the anionic component of pyrite (FeS_2_). Incidentally, selenium is a constant geochemical companion of sulfur. This means that early on any pathway to Cys-units would actually have been a twin pathway to Cys units and selenoCys (Sec) units as siblings. Later selenocysteine came to be formed and infiltrated into the process of translation by a separate pathway with a separate elongation factor SelB [106].

Two extant amino acids (Thr, Met) derive from Asp and two other extant amino acids (Pro, Arg) derive from Glu, all four pathways beginning with ATP-driven reductive conversion of the ω-carboxylate group to an ω-aldehyde group (–CO-OH → –CO-AMP → –CHO). In the case of Glu the aldehyde group then undergoes either reductive amination with the α-amino group to form proline (Pro) or transamination with Glu to form ornithine (Orn), which then is converted to arginine (Arg) via citrulline. In the case of Asp the aldehyde group undergoes further reduction to form homoserine (hSer), which sits at a metabolic branch point. In one reaction branch hSer is phosphorylated, followed by PLP-catalyzed rearrangement to Thr. In a second reaction branch hSer is converted to homocysteine (hCys), which subsequently is methylated to Met. The new amino acids Pro, Arg, Thr, Met are then charged onto dedicated tRNAs by aminoacyl-tRNA synthetases. It may be speculated that tRNA-dependent short routes preceded these long routes. All key intermediates in these pathways are amino acids that have today no other function than being intermediates. By the Wong theory the intermediate amino acids Orn, hSer, hCys, must have functioned in early forms of ribosomal translation, which poses an interesting problem. In Orn-tRNA, hSer-tRNA and hCys-tRNA the terminal H_2_N–, HO–, HS-groups would catalyze hydrolytic cleavage of the ester link by intramolecular attack. For resolving this paradox we should recall the proposal [74,83] that early-on charged minihelix tRNA precursors were complexed on one side by a class I ur-synthetase and at the same time on the other side by a class II ur-synthetase. This implies a conformational fixation of the aminoacyl group with the effect of a possible prevention of self-catalyzed ester cleavage. Conformationally more flexible peptides/proteins, however, would suffer self-cleavage at sites of Orn, hSer or hCys units. We may speculate that early-on translation operated as a continuous process (tethered to rolling circle transcription) and that long primary peptides self-cleaved into shorter peptides at the sites of the Orn, hSer or hCys units. Later on these cleavage-causing amino acids were converted into non-cleaving amino acids (citrullin, Arg, Thr and Met), while the cleavage function was taken over by initiation and termination control systems. Actually, one of the cleavage-causing amino acids (hCys) was converted into an initiation unit (Met or fMet).

The biosyntheses of all other amino acids are rather complex: α-keto-glutarate → α-keto-adipate → α-amino-adipate → Lys (10 steps); Asp → diaminopimelate → Lys (10 steps); 2-keto-propionate/butyrate → Val/Ile (4 steps), Leu (7 steps); PEP → (Phe/Tyr (siblings, 12 steps), Trp (15 steps)); ATP → His (9 steps), Lys → Pyl (pyrrolysine, 4 steps). These pathways require PLP, and their emergence presupposes an advanced process of translation. The infiltration of the process of translation with some of these amino acids may well have also involved at first the short pathway. In the case of Pyl this may be readily appreciated. In extant biochemistry two very different pathways lead to Lys, and Lys is charged by a class I and by a class II synthetase. Lys has an important function for attaching cofactors to proteins and could have been preceded in this function by Orn. The pathway to Lys via α-amino-adipate is closely related to the formation of Orn via Glu and may have the greater priority. All multi-step pathways to amino acids are the result of a complex evolution with recruitments and lateral transfers. This evolution runs parallel to the evolution of aminoacyl-tRNA synthetases and tRNAs and to the expansion of the process of translation and of the genetic code. Among the very late arrivals in this progression are Trp and His [107]. Both share an isomerase in their biosynthetic pathways [108].

**Evolution of Genetic Coding.** The extant genetic machinery employs a genetic code comprising two successive layers of discrimination [94]. The first layer of discrimination is mainly due to selective associations between bases (or base pairs) in the Acc arms of tRNAs and primitive cores of aminoacyl-tRNA synthetases [109], specifically urzymes [87]. Charging site and charging discriminators are here in close spatial vicinity. To a lesser extent the first layer of discrimination is also due to selective associations between base pairs in the AC arms or in core regions of tRNAs and the aminoacyl-tRNA synthetases [74]. The second layer of discrimination is due to associations between triplet codons of mRNAs and the anticodons of the tRNAs. We here use the term “analogue code” for the first layer of discrimination and the term “digital code” for the second layer of discrimination. For the structural association between the tRNAs and the aminoacyl-RNA synthetases we may use the metaphor “analogue-to-digital” conversion, while the ribosome/tRNA system may be termed “digital-to-analogue” converter. In line with the rule of Florkin and Granick [57,58,59] it has been suggested that the “analogue code” has evolutionary priority over the “digital code” [110] (Schimmel theorem III). The origin of translation may be identified with the advent of the “digital code”, where after both codes coevolved with the expansion of the set of amino acid pathways, the set of tRNAs and the set of aminoacyl-tRNA synthetases.

Given present evidence, the complex coevolution of pathways and code is underdetermined, but reflected to some extent by the table of codons [95]. Amino acids with precursor-successor relationships are frequently associated with codons that are separated by one base (e.g., Asp-Asn, Glu-Gln, Ser-Cys, Ser-Sec). As another regularity we frequently see sibling amino acids as neighbors in the table of codons (e.g., Asp-Glu; Val-Ile, Phe-Tyr).

**Pre-translational Coding of Peptides.** The analogue code between bases of tRNAs and the aminoacyl-tRNA synthetases must have been operative before the digital code between mRNA codons and tRNA anticodons. This means that the process of digitally programmed translation of mRNAs into proteins was preceded by a pre-translational peptide synthesis that was tRNA-guided, but not mRNA-programmed. Let us see how far we can come in retrodicting this early system of peptide synthesis.

We recall from the discussion of the peptide cycle that the earliest peptide synthesis could have involved only a relatively small number of amino acids that were accessible by carbon fixation, e.g., some or all of the amino acids of the following set: {Gly, Ala, Ser, Asp, Glu}. They would have been activated by COS as N-carboxy-aminoacyl anhydrides (NCAAs). We also recall the suggestion that the “modern” clover-leaf tRNAs must have been preceded by pre-tRNA hairpin structures [74,111]. Early on such hairpin structure would have been a short “microhelix”, akin to the extant Acc arm. It would have evolved by hairpin extension to a “minihelix” [74]. These pre-tRNAs would have been aminoacylated at their 3’-terminal 2’-OH or 3’-OH groups [74]. Primitive precursor peptides of extant aminoacyl-tRNA synthetases would have been in service of enthalpic amino acid activation and aminoacylation of the pre-tRNAs. Some of the peptides operating as primitive aminoacyl-tRNA synthetases would have served the very peptide synthesis system whence they derived—A case of autocatalytic product feedback. Other peptides with ancillary functions, e.g., for error correction, would have cooperated with the primitive synthetases, perhaps first by forming quaternary associations and later by gene fusion.

Simple side-by-side positioning of two aminoacyl-pre-tRNA hairpin structures would lead to the formation a dipeptidyl-pre-tRNA by entropic catalysis. Proper orientation would be ensured by a metallo-oligoribonucleo-peptide scaffold—A primitive form of the ribosomal PTC. In a still earlier stage this cluster may have been preceded by a mineral surface. Discrimination of the lateral associations of different pre-tRNAs would have been caused by analogue coding close to the aminoacyl moiety. Side-by-side positioning of the resulting dipeptidyl-pre-tRNA with another aminoacyl-pre-tRNA would lead to the formation of a tripeptidyl-pre-tRNA, again under the dictates of the analogue code and so forth. This peptide synthesis may have been mediated by the aminoacyl-tRNA synthetases [112,113]. In this fashion an oligopeptide would be formed and finally set free by hydrolysis. The sequence of this oligopeptide would be determined by the lateral analogue code. The pre-ribosome as scaffold for this synthetic reaction would be a metallo-oligoribonucleo-peptide particle akin to the extant ribosomal peptidyl transfer center.

The projection of peptide synthesis into an early system of pre-tRNA catalysis is supported by a number of experimental facts. It has been shown that RNA microhelices that mimic the acceptor arm of tRNAs and harbor appropriate analogue code determinants can be aminoacylated by modern aminoacyl-tRNA synthetases [114]. It has also been demonstrated that RNA minihelices corresponding to the Acc arm are capable of cooperating functionally with the elongation factor EF-Tu [115] and with the ribosome [116]. Furthermore, charged RNA minihelices have proved to be suitable for peptide bond formation by extant ribosomes [117].

A model for primordial entropic RNA catalysis of peptide formation has been tested experimentally [118]. Specifically, two RNA minihelices with charged 3’-CCA terminals were used as tRNA precursors in conjunction with an RNA tetraplex (a duplex of two RNA duplexes) with four unpaired sequences complementary to the CCA sequences of the RNA minihelices. It was determined experimentally that complexation does occur by base pairing between the unpaired sequences of the minihelices and the tetraplex. This complexation positions the minihelices side by side, but ester aminolysis does not occur for lack of proper orientation. By contrast, the extant ribosomal peptidyl transfer center does have the properly orienting profile for ester aminolysis, presumably because it appears to be not just an oligoribonucleotide, but rather a metallo-oligoribonucleo-peptide particle.

It is sometimes argued that there is no informational or structural continuity between pre-translational peptide products and translational protein products [79]. This position may well be unfounded. The sequences of pre-translational peptide products may have been controlled by the analogue code between tRNAs. With the beginning of mRNA-programmed translation the digital triplet code is initiated and superimposed over the analogue code. Thus, the emerging digital triplet code between mRNA codons and tRNA anticodons would have had to adapt to the primary analogue code. It would have subsequently evolved in close correlation with the further evolution of the analogue code as the translation process came to be infiltrated with more and more amino acids. Therefore, we may be justified to postulate a certain informational/conformational/functional continuity between peptide formation prior to the beginning of mRNA-programmed translation and later translational protein formation, *cf.* [60] *vs.* [79].

## 7. The Place of RNA in the Origin of DNA

All extant cellular organisms, and by extension LUCA, are characterized by two types of nucleic acid: RNA in charge of transient catalysis, and DNA in charge of enduring inheritance. The biosynthesis of deoxyribonucleotides for DNA proceeds without exception from ribonucleotides. In agreement with the rule of Florkin and Granick [57,58,59] it is generally concluded that ribonucleotides preceded deoxyribonucleotides in evolution. This conclusion is supported at the functional level. Deoxyribonucleotides have only one function: as monomers for the formation of DNA. Ribonucleotides serve not only as monomers, but also, importantly, as energy coupling agents and as starting materials for the biosynthesis of coenzymes and histidine. Ribonucleotide monomers serve for the formation of oligoribonucleotides or relatively short, sequence-sensitive, single-stranded RNA molecules for catalytic purposes or guide purposes. Deoxyribonucleotide monomers serve for the formation of relatively long, double-stranded DNA molecules for long-term heredity purposes. The RNA World theory postulates that early on RNA was also in charge of inheritance to be later replaced by DNA. There is no evidentiary support for this postulate. No extant cellular organisms has an RNA chromosome or plasmid. According to the FeS World theory evolution of RNA catalysts is primary. Yet, DNA must have followed on the heels of RNA for long-term preservation of emergent valuable sequence information and the force of this situation led quickly to the apotheosis of DNA.

Let us now consider the origin of DNA in detail. The conversion of ribonucleotides into deoxyribonucleotides by a radical mechanism may have occurred very early, perhaps soon after the appearance of ribonucleotides. It has been suggested that the required thiyl radicals were formed pre-enzymatically with FeS/H_2_S [119] (Follmann theorem). The deoxyribonucleotides would then have infiltrated an already existent process for the formation of oligoribonucleotides to generate mixed oligo-ribo/deoxyribo-nucleotides. Whenever a position vulnerable to self-cleavage by 2’-OH would have come to be occupied by a deoxyribonucleotide a possibility for self-cleavage would have been eliminated and the resulting co-oligomer would have acquired a correspondingly extended half-life. In this manner a natural dichotomy would have arisen between more and more stable co-oligomers with more and more deoxynucleotide units and oligoribonucleotides. Eventually this dichotomy would gravitate towards the DNA/RNA dichotomy, which is from the start a dichotomy between essentially transient catalytic RNA with half-life on a short-term metabolic time scale and hereditary DNA with half-life on an increasingly long-term geologic time scale.

Another distinctive feature of DNA, the use of 5-methyl-uracil or thymine (T) instead of its functional equivalent uracil (U), is also related to the thermo-stability problem. The base cytosine (C) undergoes facile deamination at a rate that increases with increasing temperature and increasing deviation of pH from neutrality [120]. In DNA such deamination is readily corrected by a base excision repair mechanism that distinguishes non-native U from native T and replaces U by C. RNA never had to acquire such repair mechanism, because it serves as transient catalyst with a high rate of turnover. Moreover, in RNA such deamination replaces the base pair GC by the base pair GU, which is weaker than GC but still operational for stabilizing RNA folding structures. For repairing the deamination of adenine (A) and guanine (G) the base excision repair mechanism is immediately applicable, because the deamination products hypoxanthine (H) and xanthine (X) do not occur in DNA and are readily recognizable as alien bases.

In extant metabolisms two different thymidylate synthases (ThyA, ThyX) convert 2’-deoxyuridine-5’-monophosphate (dUMP) into 2’-deoxythymidine-5’-monophosphate (dTMP) [121,122] (followed by double phosphorylation). Both enzymes use CH_2_=H_4_folate as CH_3_-source for U → T conversion. For methylations of other bases, e.g., cytosine, the CH_3_-source is S-adenosyl-methionine (AdoMet). These two methyl sources are located at the two extreme ends of a C1-reduction cascade: CH_2_=H_4_folate → CH_3_-H_4_folate → CH_3_-Co (Methylcobalamin) → Methionine → Ado-Met. By the rule of Florkin and Granick [57,58,59] we conclude that methylation with CH_2_=H_4_folate has greater antiquity than methylation with AdoMet and that it goes back to a time long before LUCA. Methylation with CH_2_=H_4_folate requires a reducing agent. ThyX utilizes an electron transport from NADPH to the flavin shuttle (FAD → FADH_2_) and from there by hydride transfer to U, thereby converting U to a U-carbanion intermediate. ThyA generates the required U-carbanion through nucleophilic attack by a Cys-S^−^ group, where after it utilizes CH_2_=H_4_folate as CH_3_-source and as reducing agent, thereby generating H_2_folate. In view of its sulfur catalysis and the obviation of a reducing chain ThyA is considered to be older than ThyX. We may even surmise that the conversion U → T emerged before the advent of AdoMet.

Early DNA must have had U as a canonical base and to this day there are viruses with uracil-DNA [123]. How could the sophisticated U → T-conversion/U-excision system have come about? A pre-existent enzyme for the excision of xanthine or hypoxanthine may have been recruited for the removal of U by discriminating faulty U-G base pairs from proper U-A base pairs. Poor U-G/U-A selectivity may have been the driving force for the U → T conversion by ThyA or by a simpler precursor thereof, which led to the more facile discrimination of faulty U-G base pairs from proper T-A base pairs. For a while there may have been competitive incorporation of U and T by dUTP and dTTP. This competition was irreversibly eliminated when the enzyme dUTPase emerged and started to purge the cell of any dUTP [123].

## 8. The Place of Peptides in the Origin of RNA

We finally approach the problem of the origin of RNA. We draw a broad context that extends from carbon fixation to RNA and from ligand-accelerated catalysis to replication and in doing so we bear in mind that biochemical reality is the intersection of functional feasibility and biosynthetic accessibility.

**Primordial Purines.** Extant RNA comprises two kinds of bases: the purines adenine (A), and guanine (G), and the pyrimidines uracil (U) and cytosine (C). They form the main base pairs A—U, G—C, and the lesser base pair G—U. Purine nucleotides, or intermediates in their biosynthesis, are starting materials for the biosynthesis of thiamine, histidine, (methano) pterins, molybdo/tungsto-pterins and flavins. Pyrimidines have no such biosynthetic function. They differ from purines by lack of an imidazole ring. Purine nucleoside triphosphates (ATP, GTP) function as universal energy coupling agents while pyrimidine nucleoside triphosphates are specialized energy couplers, UTP for sugar metabolism and CTP for lipid metabolism. The biosynthesis of purine nucleotides is essentially a piecemeal C1-fixation pathway. Pyrimidine nucleotides form by coupling pre-synthesized modules. The pyrimidine cytosine has a high propensity for deamination, while the purines are more stable. Also purines have a higher stacking energy than pyrimidines. For all these reasons evolutionary priority is attributed to purine nucleotides while pyrimidine nucleotides are seen as later inventions of life [124].

The extant system of purine pathways is universal, while differences exist in terms of enzymes and cofactors [125,126]. The first committed intermediate, 5-phosphoribosylamine, is extremely instable (half-life of 5 s) and requires channeling. Six steps of the pathway require coupling with ATP hydrolysis to generate amide, amidine or urea moieties. We therefore postulate that a much simpler precursor pathway to purines must have existed with the following characteristics:

(1) Sole dependence on volcanic-hydrothermal C1-compounds, e.g., CO, CO_2_, COS, HCN, H_2_NCN, with chemical potentials derived by quenching volcanic gas, or by hydrolysis of magmatic/crustal high temperature minerals;
(2) Dependence on transition metals (e.g., Ni) suitable for ligand-accelerated catalysis;
(3) Continuity with extant *de novo* purine pathway, notably with regard to piecemeal conversions to energy coupling with ATP;
(4) Use of stable peptidyl-amines as precursors of instable 5-phospho-ribosylamine;
(5) Exergonic formation of amide/amidine/urea moieties, e.g., from HCN or H_2_NCN.

**Figure 5:**
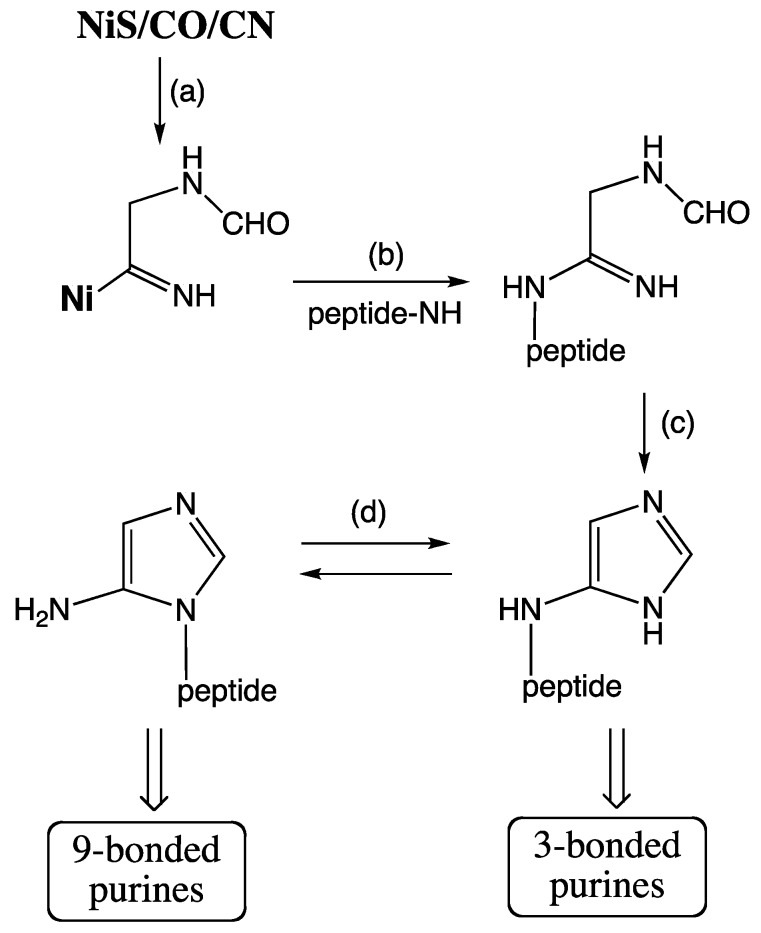
Hypothetical precursor pathway to purines.

In agreement with these characteristics a primordial precursor pathway is suggested as shown in Figure 5. An imidazole unit is generated by organo-metal carbon fixation (reactions a,b) on NiS, *cf.* [49] and by cyclization as previously demonstrated at 150 °C [127], coupled to the free amino group of a peptide. This imidazole unit is reminiscent of the terminal hydantoin ring that forms in the course of the peptide cycle (Figure 3) [49] and it may have been a functional precursor of histidine.

The pathway indicated in Figure 5 will naturally lead from an open structure by ring closure to two sibling imidazole units, wherein the peptide amino group is either endocyclic or exocyclic [124]. These may also interconvert by Dimroth rearrangement [128,129]. Subsequent annulation of the pyrimidine ring, therefore, generates two sibling purine types, one attached at position 9 of the imidazole ring and the other at position 3 of the pyrimidine ring. Imidazoles, purines and all intermediates are excellent ligands for transition metals, e.g., [130]. Their ligand function is seen as preceding their base-pairing function. Therefore, the oxidation state of the purines may range from the most oxidized uric acid via xanthine (X) to hypoxanthine (H); or their nitrogen analogues diaminopurine (daP), guanine (G), isoguanine (iG), adenine (A). The proposal is supported by the formation of purines from an (unrealistically concentrated) aqueous solution of ammonium cyanide or from non-aqueous formamide [131,132], and also by formation of glycinamide from Ni/CN/CO [46] and of a hydantoin ring in the peptide cycle (Figure 3) [49]. Thus, an early structural/functional precursor of nucleic acids was likely comprised of a short peptide with an attached N-heterocycle (a hydantoin ring, or an amino-imidazole with later annulation of a pyrimidine ring) with the ability to ligate to transition metals. This initial structure evolved into (oligo)ribonucleotides. We approach this transition by focusing first on the evolutionary precursor of the phospho-ribose moiety.

**Trionucleic acids.** The RNA World theory distinguishes between “biotic chemistry” that begins with RNA formation/replication and “pre-biotic chemistry” that supplies activated nucleotides by a “multi-pot” reaction: (1) input of formaldehyde at very low concentrations into the primitive ocean; (2) diversity of concentrating mechanisms [133]; (3) formose reaction with Ca(OH)_2_ and with boric acid [134] or cyanamide [135] for favoring ribose by group protection; (4) phosphorylation of ribose [136]; (5) condensation reaction of phospho-ribose with a pre-established mixture of bases; (6) further phosphorylation of the resulting ribonucleosides; (7) polycondensation and replication. These stages require change of reaction conditions by transport of intermediates from one reaction theatre to the other. For simplifying the problem a “one-pot” reaction scheme has been devised that generates nucleotides by concerted formation of the ribose ring and an attached pyrimidine base [137]. Aside from the problematicity of its starting materials, this ingenious approach has so far not been shown to be applicable to the all-important purine bases.

**Figure 6:**
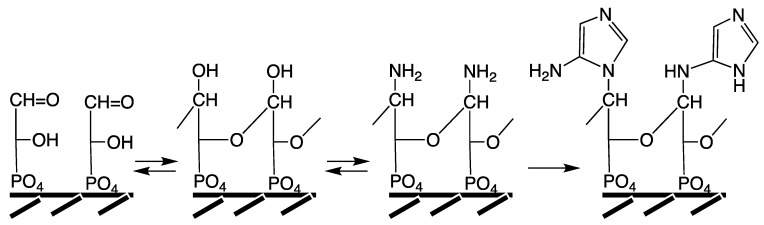
Hypothetical conversion of 3-phospho-glyceraldehyde to surface-anchored trionucleic acid with imidazole bases (representation chosen for facile perception).

For developing an alternative that is compatible with the FeS World theory we consult extant biosynthetic pathways. RNA owes its very structure to the triple-bonding ribose rings. In its biosynthesis 5-phosphoribose is converted to nucleotides via 5-phosphoribosyl-1-pyrophosphate. In autotrophic organisms 5-phospho-ribose is formed either by a salvage pathway (phosphorylation of ribose), which is judged here to be a latecomer; or *de novo* by a carbon fixation pathway. The Calvin cycle as a source for 5-phospho-ribose is also judged to be a latecomer. This leaves us with the pathway that starts with production of an acetyl thioester by the acetyl-CoA pathway and/or as key intermediate in the reductive citric acid cycle, which is actually a thioester cycle and may be the first metabolically autocatalytic cycle [44]. The acetyl thioester is then converted into pyruvate under autotrophic conditions. The subsequent pathway steps proceed today via phosphoenol pyruvate and 3-phospho-glycerate to the 3-phospho-trioses and from there by the pentose phosphate cycle to 5-phosphoribose. This pathway is clearly very complex and fully dependent on sophisticated enzymes. For simplification we apply the rule of Florkin and Granick [57,58,59] and postulate that 5-phosphoribose is predated by the two 3-phospho-trioses, 3-phospho-glyceraldehyde (PGA) and dihydroxyacetone phosphate (DHAP), which interconvert non-enzymatically via an enol intermediate [138]. Moreover, we recall the proposal that phosphate groups had the original function of serving as surface bonding mediators or ligators [42,139]. Based on these postulates we carry out a comparative thought experiment (Figure 6). Let us assume that GAP or DHAP molecules are ligated with their phosphate groups onto (transition) metal centers in or on a mineral surface (under water) and that bonded molecules have the proper general orientation and sufficient proximity for interaction. By the laws of chemistry their oxo and hydroxyl groups will then connect intermolecularly by hemiacetal (or hemiketal) bonds to form more or less extended oligomeric structures in competition with hydration [42]. In ribose such bonds are formed intramolecularly. For dissolved phosphotriose molecules the entropic burden precludes significant hemiacetal (or hemiketal) formation. However, for crystals of NH_4_-DHAP hydrate hemiketal bonds are constitutive [140]. The state of surface bonding may be seen as an intermediate state between the solution state and the solid state that gives rise to quasi-intramolecular hemiacetal/ketal bond formation. In the presence of ammonia the resulting surface-bonded oligo-hemiacetal (or oligo-hemiketal) structures are apt to be infiltrated by amino groups. These then would compete with the amino groups of peptides to serve as anchors for the formation of attached amino-imidazole groups, and subsequently purines (Figure 6). These hypothetical polymers, termed “trionucleic acids”, would be the ultimate precursors of ribonucleic acids [42].

The formation of surface-bonded trionucleic acids is thermodynamically driven and avoids arduous multi-step pathways. Key assumptions of the thought experiment are supported by experimental demonstration of the formation of glyceramide and glycerate by carbon fixation in the aqueous system NiS/CO/CN [49]. Primordial reduction of 3-phospho-glycerate to 3-phospho-glyceraldehyde is supported by the tungstopterin-catalyzed reduction of non-derivatized –COOH groups to –CHO groups [141]; and by the experimentally supported conversion of –COSH to –CHO [142]. As source for phosphorylation volcanic tetraphosphorus decaoxide (P_4_O_10_) or products of its partial hydrolysis or aminolysis [143,144] may be considered. Alternatively phosphorylation agents may derive from a nucleophilic attack of adsorbed phosphate ions [145] on acetyl-thioester [146] that forms by Ni-catalyzed carbonylation of volcanic methylmercaptan [54], or on N-carboxyaminoacyl anhydrides that form by activating amino acids with COS [52]. The thermostability of 3-phospho-glyceraldehyde phosphate (or dihydroxyacetone phosphate) in aqueous solution is low, but (at 70 °C!) it was shown to be increased 5-fold (or 8-fold) by the presence of Fe^2+^ ions [147]. It would increase further by formation of mineral-supported hemiacetal (or hemiketal) structures or their analogues with pendant amino groups.

The mineral-anchored trionucleic acids are seen as breeder structures for the emergence of base pairing within the context of transition metal ligation. Throughout the development from amino-imidazoles to purines free electron pairs of the heterocycles are available for ligation [130]. The imidazoles have strong ligation properties, but low stacking affinity and essentially no base pairing ability. The emergent purine bases still exhibit ligation ability, but in addition also strong stacking affinity to each other and the ability to form base pairs. It is suggested that base stacking emerged first and that base pairing by hydrogen bonds followed later. Throughout this transformation the surface-anchored trionucleic acid (Figure 6) is expected to have a dynamic structure, subject to alternation between integration and desintegration of the hemiacetal/ketal bridges. The rate of chain breakup would depend on temperature, on pH and on the structure of the heterocycle moiety. Yet, overall the ligand effect by imidazole/purine bases would persist as long as the chemical conditions permit their continued re-synthesis. By this reaction dynamics the surface-anchored trionucleic acid structure is self-selecting towards increased stability and increased ligand function (ligand accelerated catalysis). In this sense trionucleic acids display base reproduction by synthesis as mechanism for sequence variation and selection, a rudimentary form of genetic evolution. Thus, early on metabolic evolution and incipient genetic evolution coincide in one pre-dichotomous, dynamic chemotype.

**Figure 7:**
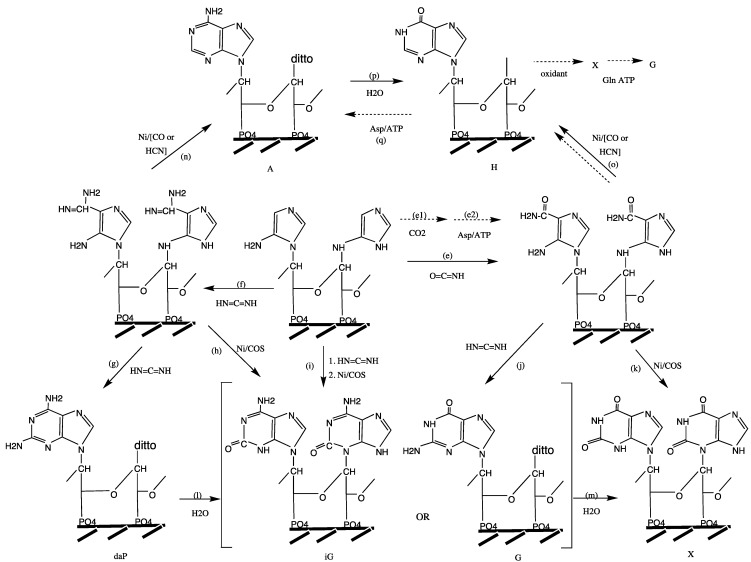
Formation of purine bases on trionucleic acid (doted arrows: endergonic pathways akin to extant *de novo* purine biosynthesis).

**Origin of Base Pairing.** We now turn specifically to the formation of purine bases by annulation of a pyrimidine ring onto the pre-synthetized imidazole ring (Figure 7). The extant annulation pathway uses low energy nutrients (carboxylation (e1), amidation with Asp (e2, q), formylation (o)) and it requires energy coupling. It is of interest for the following discussion that structural phylogenomic studies of the purine pathway reflect a piecemeal enzymatization of an archaic pathway [148]. Here a primordial pathway is suggested that follows the same basic course as the extant pathway. It proceeds through C1-condensations (e to k, n, o) and deaminations (l, m, p) and it is exergonic without energy coupling due to the use of sufficiently energetic nutrients: O=C=NH (e), H_2_NCN (f, g, i, j), Ni/COS (h, i, k) and Ni/CO, CN (n, o). Incidentally, the carboxylation step (e1) has been demonstrated experimentally for a model compound in hot, saturated aqueous KHCO_3_ [127].

The structure of trionucleic acid, shown in Figure 5, Figure 6 and Figure 7, is based on 3-phospho-glyceraldehyde, with two chirality centers. They may also comprise dihydroxyacetone phosphate units with one chirality center. This opens up a large number of different backbone structures. Since there are no rings involved, the structures are quite flexible and may exhibit a large variety of conformations dependent on the type of mineral surface. Presence of 9- and 3-bonded purines gives rise to purine-purine base pairing with Watson-Crick geometry between 9- and 3-bonded purines (9A--3X, 9H--3daP, 9H--3iG, 9G—3iG, 9daP--3X) [124]. By this base-pairing strand segments of ribonucleic acid may combine to form two-dimensional surface-bonded arrays. In these arrays base pairing in conjunction with surface bonding is expected to force configurational and conformational regularity. The base pairs may be oriented in parallel to the mineral surface (stacking to the mineral surface) or normal (or oblique) thereto with purine-purine stacking. In a syntactic structure all bases are oriented to the same side with spacing equivalent to three single backbone bonds, suitable for a high stacking energy. Two such strands may be oriented antiparallel to each other and form a ribbon structure. This means that base pairing would be strengthened by base stacking and force homochirality within each strand. In a syndiotactic structure bases are oriented alternately to one side or the other with spacing equivalent to six single backbone bonds, and base pairing would generate an extended multi-strand array structure, again with forced regional homochirality.

**Transition to RNA.** Evolution from trionucleic acid to ribonucleic acid had to span a seemingly large structural distance with transformations of several structural/functional features: (a) replacement of triose skeletons by ribose rings; (b) relocation of the glycosylamine moiety into the ribose ring; (c) replacement of phospho-monoester moieties by phospho-diester bridges between ribose rings; (d) changeover to 5-phospho-ribose-1-pyrophosphate; (e) functional replacement of the dynamic behavior of trionucleic acid by the self-cleavage ability of RNA.

Proceeding in the retrodictive direction we are faced with a fundamental alternative regarding the starting platform. Is phospho-ribose in RNA a biochemical singularity, imposed by intrinsic structural/functional/biosynthetic restrictions—or was it selected from a range of coexisting and competing alternatives? This question has been pursued systematically by Eschenmoser with the synthesis of nucleic acids having alternative phospho-sugar backbones [149]. Aldohexose-based nucleic acids are not capable of informational base pairing. Base pairing of the erythro(furanosyl)nucleic acid is sterically impossible. Threo(furanosyl)nucleic acid (threo-NA) exhibits strong informational Watson-Crick base-pairing with itself and with RNA. It lacks, however, a vicinal free hydroxyl group and so it has no self-cleavage ability as required for a catalytic nucleic acid. Therefore, threo-NA could not function as an RNA precursor. This leaves us with the four aldopentoses ribose, lyxose, xylose, arabinose. The four (4’ → 2’)-linked pentopyrano-NAs show the ability for strong Watson-Crick base-pairing in the form of single-strand hairpin folding structures or antiparallel double helix structures. They have, however, no ability to form informational hybrid base-pairing with RNA or DNA. In addition, the (4’ → 2’)-linked xylopyrano-NA has no ability for self-cleavage. The other three (4’ → 2’)-linked pentopyrano-NAs are neither sufficiently self-cleaving to serve as RNA precursors, nor sufficiently stable to serve as DNA precursor. Moreover, these structures could not have operated as competitors of catalytic RNA for reasons of excessive duplex stability (rigidity). Of the four (4’ → 3’)-linked pentopyranosyl structures informational Watson-Crick base-pairing is not observed for ribose due to repulsion of the vicinal phosphodiester groups and the same negative result is to be expected for xylose and arabinose. The (4’ → 3’)-linked lyxopyranosyl structure, which lacks vicinal repulsion, shows Watson-Crick base pairing, but only to a weak extent. It is also ruled out. This leaves us with the narrow class of pentofuranosyl structures to which RNA and DNA belong. Arabinofuranose has the same cis or “axial” 5’C-to-3’OH configuration as RNA. Hence it also is capable of informational base pairing with itself and with RNA [150,151]. However, its 2’-OH is trans to 3’-OH. Therefore, arabinofurano-NA is not self-cleaving and must be ruled out for that reason. Xylofuranose and lyxofuranose show trans configuration in their 5’C-to-3’OH moiety and so they are also ruled out. This leaves us with ribofuranose as the sole basis for a functional nucleic acid system (Eschenmoser theorem).

Biosynthetic conversion of 3-phospho-glyceraldehyde and dihydroxy-acetone phosphate into fructose 1,6-bisphosphate proceeds either by a Schiff base mechanism or by divalent transition metal catalysis. Subsequently, fructose-6-phosphate forms by hydrolysis and enters a system of near-equilibrium transaldolase/transketolase reactions that involve 3-phospho-glyceraldehyde, 4-phospho-erythrose, 5-phospho-ribose, 5-phospho-xylulose, 6-phospho-fructose and 7-phospho-sedoheptulose in coexistence. Surprisingly, these constituents interconvert in anoxic aqueous solutions at 70–90 °C (not below 40 °C) in the presence of dissolved Fe^2+^, Co^2+^ and Ni^2+^ ions [147]. These results, as well as the demonstration of enzyme-free, CN-catalyzed transaldolase conversion of fructose + glyceraldehyde into xylulose + erythrose [152] suggest that the conversion of 3-phospho-triose into 5-phospho-ribose would have proceeded under conditions of the FeS World theory (Breslow-Ralser theorem).

Let us now see how these conversions may fare in a mineral-supported autotrophic metabolism. We apply the principle of biochemical continuity and the principle of minimum structural change [153] and surmise that the conversion of 3-phospho-triose into 5-phospho-ribose occurred as part of the conversion of trionucleic acid into ribonucleic acid. This transition requires a changeover from phospho-monoester units to the phospho-bisester bridges, which may have been promoted by primordial phosphorylation agents, e.g., volcanic P_4_O_10_. The changeover must have occurred in a state of surface bonding. At this stage a chemical selection takes place. Structures with proper configuration and base sequence come free from the mineral surface to form self-supporting oligoribonucleotides by base stacking and by ligation to divalent cations, like Mg^2+^. Later, with the advent of pyrimidine nucleotides the 3-bonded purines would have been gradually replaced by essentially isogeometric and isoelectronic pyrimidines (3-X ⇒ 9-U; 3-iG ⇒ 9-C), which completed the conversion to RNA.

**Origin of Coded Polycondensation.** In the course of lift-off of some of the oligoribonucleotides the loss of mineral scaffold support would have been compensated by peptide scaffold support, generating metallo-oligoribonucleo-peptide particles. A crucial structural observation supports this view [154]. The double helix conformation of a β-sheet of two antiparallel peptides fits precisely into the minor groove of an RNA double helix. Based on this finding by structural modeling it was suggested that early on a mutual scaffolding effect was at work, an RNA double helix serving as catalytic scaffold surface for peptide synthesis from activated amino acids, and reciprocally, a peptide β-sheet double helix serving as catalytic scaffold surface for RNA synthesis from activated nucleotides. Aminoacyl-nucleotides (or perhaps a NCAA-NMP combination) may have served as a unitary monomer system of for both polycondensations. This indirectly autocatalytic mutual scaffolding has been suggested as a rudimentary form of coding with sufficient looseness to permit not only “mutations” and selection, but also an intrinsic route to chain extension. In addition, integration of oligoribonucleotides and peptides to composite particles would have lent added thermal stability to both the peptides and the RNAs. The suggested chemical system displays a stunning degree of backward convergence of RNA-“coded” peptide formation and peptide-“coded” ribonucleotide formation into one unitary process (Carter-Kraut theorem). 

**Origin of Homochirality.** As a consequence of the Carter-Kraut theorem mutual catalysis means homochirality within each polymer strand and in addition a chiral complementarity between l-amino acid peptides and d-oligoribonucleotides or between d-amino acid peptides and l-oligo-ribonucleotides, whereby both types jointly reflect the overall racemic states of d,l-amino acids and d,l-ribonucleotides. Specifically, an all-d RNA helix scaffold would select l-amino acids for polycondensation to an all-L peptide β-sheet. By the same token an all-l peptide β-sheet scaffold would select d-ribonucleotides for polycondensation to an all-d RNA helix. Conversely, a mutual selection of all-l RNA and all-d peptides would also have to occur to maintain an overall racemic state.

It is widely held that the changeover to universal homochirality (symmetry breaking) was a result of two consecutive mechanisms: (1) formation of a deviation from the racemic state (enantiomeric excess); (2) amplification to homochirality by autocatalytic effects [155]. Chiral segregation of extended oligomers (regional homochirality), first on a mineral surface, e.g., a pyrite surface [43], and later in accordance with the Carter-Kraut theorem, may have ushered the process along by replacing (as target of amplification) a system of dissolved small molecules by a system of oligomers, with mineral support and/or mutually supporting peptide-RNA oligomers. In the case of mineral support the original enantiomeric excess may have been inherited from an enantiomeric excess of a supporting chiral mineral, e.g., pyrite, which in turn may be determined by compounded parity-violating energy differences of plural structural units, either of the full crystal structure or of a surface layer thereof [43,156,157,158].

## 9. The Place of a Structured Flow Setting for the Origin of Genetics

Metabolic processes are driven by chemical potentials. Continuance of these chemical potentials requires transport of nutrients and waste products relative to the site of the metabolism. This notion of transport presupposes a heterogeneous relationship and a relative flow between an organism and its aqueous environment. Prior to cellularization only a mineral could have provided the required organism-environment dichotomy. In the RNA World context nucleotides of unknown origin exhibit transport by diffusion in a prebiotic broth until they are adsorbed by a mineral surface. Thereby they increase their effective concentration for interaction. In the FeS World context organic compounds are generated in a surface metabolism by *in situ* carbon fixation and by surface bonding *in statu nascendi*, catalyzed by transition metal centers and driven by chemical potentials of quenched volcanic-hydrothermal nutrients. By the logic of maintaining chemical potentials the “original homestead of life” was defined as “a place with liquid water having a nearly neutral pH where hot volcanic exhalations clash with a circulating hydrothermal water flow” [42]. Increase of the specific surface area by porosity is equivalent to an increase of surface-bonding sites and catalytic activity for the surface metabolism. The surface metabolism in conjunction with flow condition amounts to “reaction-chromatographic” behavior with continuous input of starting material and differential retention of reaction products [22,23]. In this context mineral pores in the nm range provide a molecular sieve effect that affects the differential retention times. In this regard it is of important that open-pore mineral aggregates with narrow passageways between pores have been invoked for size exclusion chromatography with separation of RNA molecules [111] (Kuhn theorem).

Regardless of theoretical context, fatty acid lipids must have had a special role in the origin of life. According to the RNA World theory they would have accumulated in the ocean, and after reaching the required concentration they would have formed vesicles. According to the FeS World theory they would have been formed *in situ* by carbon fixation to subsequently travel with retention times that increase with increasing chain length. Their first function would have been differential lipophilization of the mineral surfaces thereby decreasing the propensity for hydrolysis and increasing the propensity for condensation reactions, including the condensation reactions that gave rise to lipids [42]. With increasing chain length a state is invariably reached when the surface-bonded lipids organize into surface-bonded membranes (monolayer, bilayer or interdigitated). At the next stage a surface bonded membrane would detach locally as a blister (“semicell”), partly bounded by the mineral surface and partly by the membrane, and harboring a pre-cytosol. At that stage a cytosol metabolism would commence and the incipient genetic machinery would make the decisive turn to a cytosol-born multi-component system. Moreover, chemiosmosis would begin with cross-membrane transport of cations, notably H^+^ or Na^+^ that coupled an exergonic redox reaction with endergonic redox reaction [159]. It may at that early stage have already involved endergonic-exergonic electron bifurcation [160].

An ingenious, experiment-based “chemical garden” proposal sees early evolution inside a mound of open FeS-bounded “cells”. A FeS-membrane precipitated at the top of the mound, where hot, alkaline, sulfidic vent fluid contacted warm, acidic, carbonated ocean water with dissolved Fe^2+^. The mound grew by breaking and remaking of the FeS-membrane. A pH gradient across the top FeS-membrane fuelled the synthetic reactions inside the mound by chemiosmotically driven ATP synthesis. As the mound grew in height the metabolism inside the open-cell structure evolved from FeS-catalyzed carbon fixation at the bottom via the RNA world in the centre to LUCA higher up—with full-fledged cells of bacteria and archaea breaking loose at the top [161]. Unfortunately, however, the experimentally detected cell structure is a freeze-drying artefact [22]; RNA cannot exist under hot alkaline conditions; and an experimental FeS membrane “behaved more like a permeable reactive barrier than a membrane” [156].

Volcanic-hydrothermal flow ducts for the origin of life must have existed in many locations and by the invariant laws of chemistry autotrophic reactions must have been essentially the same everywhere. Later, locally different varieties with different catalytic endowments would have arisen. At that stage global uniformity of life would have been maintained by large impactors that ejected crustal material high into the atmosphere or even into orbit. Inside these ejected junks of crustal material catalytic/genetic endowments (first transition metal complexes with more or less evolved organic ligands, later systems with nucleic acids and finally whole organisms) would have survived for subsequent scattered reentry [162] (Cockell theorem). Fluid flow through a localized volcanic-hydrothermal flow duct is of a short duration, too short to cover a long period of evolution. Intense ejection and scattering of crustal materials, however, and their settlement in renewed flow beds would cause the process to be resumed time and time again with ever more advanced catalytic endowments and cellular organization.

## 10. Conclusions

According to the RNA World theory the process of early evolution is genetic and stochastic from the start and all of its problems may be encapsulated by the notion of an “order-out-of-chaos” self-organization, which does not avail itself to a progressive research program. According to the FeS World theory the overall process of early evolution runs along two tracks. The primary track of evolution is a chemically pre-determined process beginning with a uniquely privileged set of catalytic transition metals and nutrients and with synthetic carbon fixation reactions directly generating small organic ligands for more advanced metallo-catalysts. Later this primary process of direct evolution shoulders a secondary, genetic process of indirect evolution. Modified polynucleotide sequences are translated into new peptides and these then become ligands for more advanced metallo-catalysts. This secondary genetic track is stochastic and requires mutations and selection with a tempo of evolution that is greatly promoted by elevated temperatures [163] (Wolfenden theorem III). So, the genetic machinery turns out to be the great randomizer of life, invented by life itself for the purpose of adaptation. Evolution becomes more and more dominated by this genetic track, so that today we have the impression that it is the only track. The primary track, however, continued, with crucial punctuations of chemical predetermination, first mainly by the intrinsic (bio)chemical predeterminations of metabolic change and lipid membrane segregation, and later by extrinsic (geo)chemical predeterminations of selection pressures for adaptation. This dual track evolution may be characterized by the slogan “order out of order out of order”. The initial order is the order of the universal laws of chemistry and it is preserved to this day by cores of chemical invariance that are locked within structures and processes of central biochemistry and surrounded peripherally by products of genetic variability [164]. Nothing in early evolution makes sense except in the light of underlying bouts of chemical predetermination.
